# Chemical Composition and Biological Activities of Diverse Products from *Commiphora gileadensis*: A Comparative Review

**DOI:** 10.3390/ph19030391

**Published:** 2026-02-28

**Authors:** Fawaz K. Alanazi, Nashwa Hashad, Asmaa A. Ahmed, Haitham A. Ibrahim, Reham R. Ibrahim, Mohamed I. S. Abdelhady, Eman G. Haggag, Fatma M. Abdel Bar

**Affiliations:** 1 Department of Pharmacognosy, Faculty of Pharmacy, Capital University (Formerly Helwan University), Cairo 11795, Egypt; ph.fawaz@hotmail.com (F.K.A.); nashwa.elsayed@pharm.helwan.edu.eg (N.H.); haitham_ali01@pharm.helwan.edu.eg (H.A.I.); reham_bassyoni@pharm.helwan.edu.eg (R.R.I.); mohamed_abdelhady@pharm.helwan.edu.eg (M.I.S.A.); eman.g.haggag@pharm.helwan.edu.eg (E.G.H.); 2 Department of Pharmacology and Toxicology, Faculty of Pharmacy, Capital University (Formerly Helwan University), Cairo 11795, Egypt; dr.asmaa_ali@pharm.helwan.edu.eg; 3 Department of Pharmacognosy, College of Pharmacy, Prince Sattam Bin Abdulaziz University, Al-Kharj 11942, Saudi Arabia; 4 Department of Pharmacognosy, Faculty of Pharmacy, Mansoura University, Mansoura 35516, Egypt

**Keywords:** *Commiphora gileadensis*, Balm of Gilead, *Arabian balsam*, Bisham, phytochemistry, terpenoids, biological activities, essential oil chemotypes, resin, oleogum resin

## Abstract

**Background/Objectives:** *Commiphora gileadensis* (Balm of Gilead) is an aromatic medicinal plant with a history of traditional use in ancient and Arabic medicine. It has been used traditionally to treat inflammation, infections, and wounds. Despite its long-standing cultural and economic importance, modern pharmacological validation requires a comprehensive synthesis of current scientific data. This review aims to provide a thorough comparative summary of the phytochemical composition and biological activities of its diverse products. **Methods:** An updated literature search was conducted using databases such as ScienceDirect, PubMed, Scopus, and Google Scholar, covering publications from approximately 2000 to 2025. The review included English-language peer-reviewed articles, books, and reports providing phytochemical analyses or biological evaluations. Data were manually extracted and categorized by plant parts (resin, leaves, bark, stems), major constituents, and specific pharmacological activities. **Results:** The review identified ten diverse chemical groups, mainly terpenoids (mono-, sesqui-, di-, and triterpenes) and flavonoids. Other remarkable classes included phenolic acids, phytosterols, lignans, coumarins, and fatty acids. However, the essential oil chemical profile is highly variable, influenced by geographical origin and preparation technique. Pharmacological studies demonstrated a wide spectrum of bioactivities, in particular antioxidant, anti-inflammatory, antimicrobial, anticancer, antidiabetic, and wound-healing properties. Toxicological studies classified the plant as generally non-toxic; however, there is a notable lack of clinical and pharmacokinetic data. **Conclusions:** *C. gileadensis* possesses a rich and diverse secondary metabolite profile, validating its traditional ethnobotanical applications. Future research should prioritize pre-clinical and clinical trials to establish its safety, bioavailability, and metabolic fate for its successful integration into modern medicine.

## 1. Introduction

Plants provide invaluable resources to humanity. They hold a valuable treasure of secondary metabolites that have helped save lives since ancient times. Over the years, numerous applications of natural products have been identified and developed for human benefit [1]. At present, they represent a major and indispensable source for the discovery and development of new therapeutic agents. Thousands of studies have investigated phytoconstituents and their biological effects on human health, particularly in comparison to synthetic compounds, which may be associated with adverse effects alongside their intended pharmacological benefits [2,3].

The genus *Commiphora* is part of the family Burseraceae. It includes about 150 to 200 species found mainly in tropical and subtropical areas, such as Africa, Arabia, India, and some regions of South America. This genus is characterized by small trees or shrubs with spiny branches. Their barks release aromatic oleogum resins with characteristic smells [4,5,6]. The *Commiphora* genus has been known since ancient times for its various healing properties, economic uses, and distinct botanical traits [7]. Additionally, the resins are useful for embalming, incense, and perfumery [4,5,8,9]. Since ancient times, plants of this genus have been used to treat various ailments, including inflammation, arthritis, wounds, obesity, diabetes, hyperlipidemia, and infections. These traditional applications have been validated by modern pharmacological research, which demonstrated anti-inflammatory, antioxidant, antimicrobial, hypolipidemic, and anticancer properties, along with the identification of more than 300 secondary metabolites [4,5,6,9].

Significantly, *C. gileadensis* (L.) C.Ch. is a well-known aromatic and medicinal plant in the *Commiphora* genus with a long history and several different names documented in the literature. The species name “*gileadensis*” and the popular common name “Balm of Gilead” refer to the ancient Gilead region east of the Jordan River [10]. Other popular names include “Balm of Makkah,” which is commonly used in Saudi Arabia and the Arabian Peninsula; “Bisham” and “Basin,” which are regional names frequently used in Arabic-speaking areas; “Mecca Balsam,” which is sometimes used in English-language sources [11], and Apharsemon in Hebrew [12]. These names reflect the historical use of the plant in perfumery, medicine, and religious rituals. In addition, *C. opobalsamum* and *Balsamodendron opobalsamum* are widely recognized synonyms [13]. In desert folklore, *C. gileadensis* is known as a “weather predictor” plant. Its growth and blooming cycles are traditionally used to predict rainfall and changes in weather patterns; this phenomenon is thought to be more prominent in this species than in other members of the genus *Commiphora* [14].

The resin of *C. gileadensis* was famous in ancient Arabia and the Middle East for its opulent fragrance as well as medicinal and therapeutic properties. It was a major constituent in incense and perfumes, contributing to its economic and cultural importance for thousands of years. Its essential oil and resin were used to prepare aromatic products that were traded and valued by various communities [14]. It contains a complex mixture of phenolic compounds, essential oils, and other phytochemicals, which potentially contribute to a broad spectrum of traditional therapeutic applications, including skin, liver, digestive, inflammatory, infectious, and even cancerous conditions, supported by evidence of antioxidant, antimicrobial, and hepatoprotective activities [9,15]. The primary methods of traditional use include topical application, oral administration, and inhalation [10,11,13,16,17].

Due to overharvesting and increased aridification brought on by climate change, the species is in danger of going extinct due to a sharp decline in population and loss of genetic diversity. This underscores the necessity of conservation strategies like controlled harvesting, controlled grazing, water management, and seed bank preservation [18].

*C. gileadensis* produces both resinous and non-resinous components, which are chemically characterized by many studies as attempts to understand their composition and bioactivity. The oleogum resin is rich in terpenoids, including cycloartane derivatives, which are key metabolites identified by NMR-based metabolomics, distinguishing it from other *Commiphora* species [19]. It also contains volatile compounds, such as α-pinene, sabinene, β-pinene, and *p*-cymene, which contribute to its characteristic intense, terpenic, and lemony aroma, as well as biological activities, including anti-inflammatory and wound healing effects [20]. Non-resinous components include amino acids, organic acids, sugars, phenolic acids, and polyphenols, which are also present in the cell sap and contribute to the pharmacological profile of the plant [19].

This review aims to present a thorough and comparative summary of the biological activities and phytochemical composition of resinous and non-resinous components of *C. gileadensis*. It aims to highlight the similarities and differences in their bioactive constituents and pharmacological potential by integrating available data.

## 2. Materials and Methods

### 2.1. Literature Search Strategy

An updated comprehensive literature search was conducted using electronic databases, including ScienceDirect, PubMed, Scopus, Google Scholar, and Web of Science. The search extended over the period from 1997 to 2025 and included peer-reviewed journal articles, reviews, books, theses, and authoritative reports. The following Boolean search query was applied (with minor syntax adjustments depending on database requirements): (“*Commiphora gileadensis*” OR “*C. gileadensis*” OR “*Commiphora opobalsamum*” OR “*C. opobalsamum*” OR “*Balsamodendron opobalsamum*” OR “Balm of Gilead” OR “Mecca Balsam” OR “Bisham”) AND (“phytochemistry” OR “chemical composition” OR “secondary metabolites” OR “essential oil*” OR “volatile oil*” OR “terpen*” OR “sesquiterpen*” OR “monoterpen*” OR “triterpen*” OR “flavonoid*” OR “alkaloid*” OR “resin” OR “oleogum resin” OR “leaves” OR “stems” OR “barks”) AND (“biological activity” OR “pharmacological activity” OR “antioxidant” OR “anti-inflammatory” OR “antimicrobial” OR “antibacterial” OR “anticancer” OR “cytotoxic*” OR “antidiabetic” OR “wound healing” OR “toxicity” OR “pharmacokinetic*”). Searches were performed using the default settings of each database. Although no formal field restriction was applied, emphasis during screening was placed on studies in which the key terms appeared in the Title, Abstract, or Keywords to enhance specificity. Chemical Abstracts Service (CAS) registry numbers were obtained through searches conducted in the Reaxys database (Elsevier), using compound names and structural information to ensure accurate matching [21].

### 2.2. Inclusion and Exclusion Criteria

Only English-language publications that provided phytochemical analyses and/or biological evaluations (in vitro or in vivo) of *C. gileadensis* were included. Articles that addressed other *Commiphora* species without comparative data on *C. gileadensis*, lacked methodological rigor, or were duplicate reports were excluded.

### 2.3. Data Extraction and Synthesis

Relevant data were manually extracted, tabulated, and categorized based on plant part studied (resin, leaves, bark, stems), major phytochemical constituents (e.g., terpenes, flavonoids, alkaloids), biological activities (e.g., antioxidant, antimicrobial, anti-inflammatory, cytotoxic), extraction and analytical methods used, and traditional and ethnopharmacological uses. Comparative tables were designed to highlight similarities and distinctions among plant parts. Emphasis was placed on studies employing validated experimental models and analytical tools such as GC-MS, LC-MS, HPLC, and NMR. Tentatively identified compounds of likely synthetic origin were excluded.

## 3. Botanical and Taxonomical Characteristics

Burseraceae is a family of flowering plants belonging to the order Sapindales and composed of about 18 genera comprising 700 species of resinous trees and shrubs [22]. Several of its species are economically important for their fragrant resins.

*Commiphora* Jacq is the most species-rich genus in the family Burseraceae, comprising approximately 190 species, 21 of which are listed in the ‘Red List of Threatened Species’ of The International Union for Conservation of Nature (IUCN) [23]. They are small deciduous trees or shrubs with short, often thorny branches and papery bark. The leaves are usually compound, and the plants can be identified by their resin canals and distinctive seed morphology. Morphological and anatomical features, such as the presence or absence of spines and axial parenchyma, are used to differentiate species within the genus [4,17,24].

**Figure 1:**
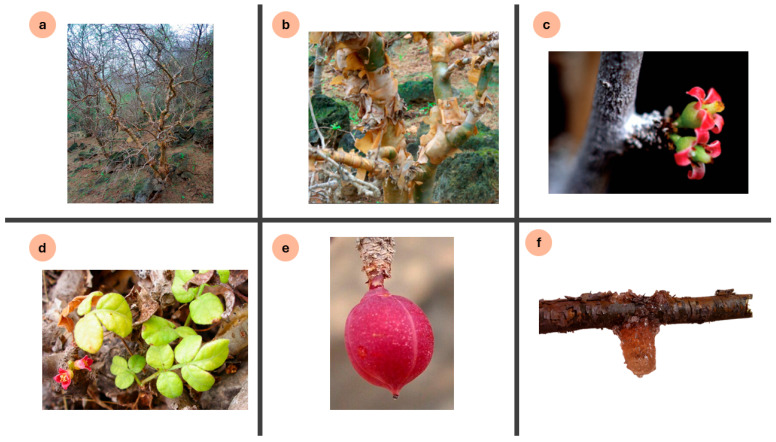
Morphological characteristics of major plant parts and products of *Commiphora gileadensis*: (**a**) whole tree and (**b**) bark [27]; (**c**) flowers and (**d**) leaves [14]; (**e**) fruit [28]; and (**f**) resin [29].

*C. gileadensis* (L.) C.Chr. is a small perennial tree or shrub, native to the southern and western mountains of the Arabian Peninsula. It belongs to the Kingdom Plantae, Phylum Angiosperms, Class Eudicots, Order Sapindales, and Family Burseraceae, and is classified within the Genus *Commiphora* [25]. It is a dioecious plant bearing scaly, dark-grey branches and small pubescent compound leaves with 3–5 leaflets (Figure 1). The flowers range from cream to red colored. The fruits are edible, red single-seeded drupes with black seeds and with white dividing lines creating the appearance of being four-lobed fruits [14]. When the bark is incised, it exudes a glossy dark brown to black fragrant oleogum resin, a key characteristic feature for the plant. Its best geological conditions are the eroded black basalt and granite rocks, with an elevation range of about 100 to 750 m above sea level, and it was not recorded on coastal plains with deep soil [17,26].

## 4. Phytochemical Profile of *C. gileadensis*

*C. gileadensis* has been the subject of extensive phytochemical research, which has consistently demonstrated that the plant is remarkably rich in secondary metabolites, particularly flavonoids and terpenes, to which a wide spectrum of pharmacological activities is potentially attributed. The phytochemical profile of the plant (Table 1) was represented by 10 chemically distinct groups, dominated by terpenoids, especially mono-, sesqui-, di-, and triterpenes, which were found in a wide array of plant material, including aerial parts, leaves, stems, fruits, bark, and particularly the resin/balsam.

### 4.1. Terpenes and Terpenoids

#### 4.1.1. Monoterpenes

**Figure 2:**
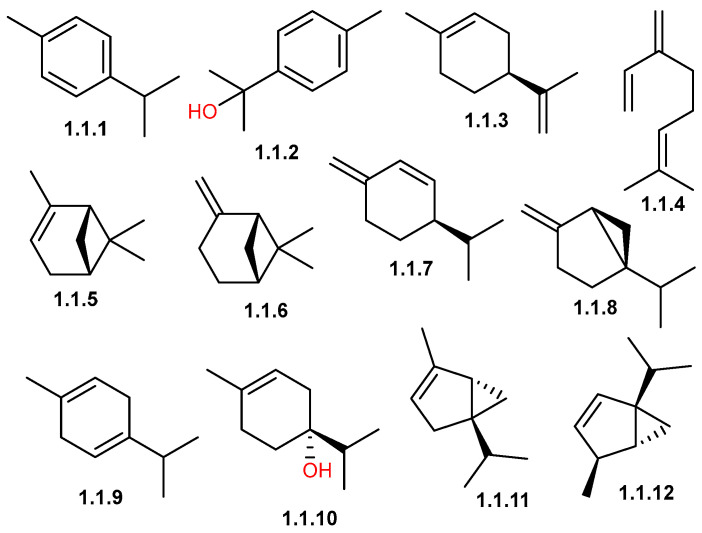
Major monoterpenes reported for *C. gileadensis* (**1.1.1–1.1.12**).

Monoterpenes are a class of natural compounds derived from two linked isoprene units, giving them a basic structure of ten carbon atoms (C_10_H_16_) [50,51,52]. Monoterpenoids (denoted by the suffix “-oid”) are monoterpenes with additional functional groups such as alcohol and ketone. Both groups are widely found in the essential oils of aromatic plants, where they often form the majority of volatile components [50,51,53]. *C. gileadensis* is rich in monoterpenoids, which constitute the dominant components of essential oils in specific chemotypes and are primarily responsible for defining its characteristic aroma profile (Table 1 and Table 2). Compounds **1.1.1–1.12** (Figure 2) represent the fundamental monoterpene profile of *C. gileadensis* in which monoterpenes hydrocarbon drive volatility and aroma intensity, while oxygenated monoterpenoids enhance chemical stability and biological relevance [54]. Monoterpene hydrocarbons, including β-myrcene (**1.1.4**), sabinene (**1.1.8**), α-pinene (**1.1.5**), β-pinene (**1.1.6**), β-phellandrene (**1.1.7**), γ-terpinene (**1.1.9**), and β-thujene (**1.1.12**), are the primary contributors to the fresh, resinous, and citrus-peppery aroma of the oil [54]. For instance, β-myrcene predominated in EO from Medina (Badr) samples, reaching 17.44%, accompanied by β-phellandrene (9.59%), indicating a monoterpene-hydrocarbon-rich chemotype [11]. In contrast, EO from plants cultivated in the Middle East but native to Saudi Arabia show strong domination of sabinene (22.7%) and α-pinene (14.4%) in fresh aerial parts, while resin and exudates are interestingly dominated by sabinene (43.8–46.4%) and α-pinene (24.0–25.8%), defining the characteristic balsamic aroma [20,54]. Aromatic monoterpenes such as *p*-cymene (**1.1.1**) serve as background components that stabilize the warm aromatic character of the oil and frequently co-occur with resin and aerial-part profiles [54]. Its oxygenated derivative, *p*-cymen-8-ol (**1.1.2**), was mainly reported in extracts and essential oils of aerial parts, enriching the oxygenated fraction [12,32]. Oxygenated monoterpenes, particularly terpinen-4-ol (**1.1.10**), are key EO components, reaching 8.5–9.8% in EOs from fresh aerial parts and flowering tops of plants from Makkah and 18.7% in oils from Almog, Palestine [20,33]. Additional contributors of this group, including limonene (**1.1.3**) and γ-terpinene (**1.1.9**), provide citrus-fresh nuances and act as biosynthetic or oxidative precursors to oxygenated derivatives [32,54].

#### 4.1.2. Sesquiterpenes

Sesquiterpenes is a diverse class of natural compounds made up of three isoprene units, resulting in a 15-carbon backbone. They are widely distributed in plants, marine organisms, and microbes, and are considered the most structurally varied group among terpenoids. The basic structure of sesquiterpenes can be acyclic, monocyclic, bicyclic, tricyclic, or even multicyclic, and they often include derivatives such as alcohols, ketones, lactones, and epoxides (sesquiterpenoids) [55,56]. Available data suggests that the sesquiterpene group is the most abundant group of compounds reported from *C. gileadensis*, especially in the aerial parts and resin, with only a few reported from the oil (Table 1). Among these compounds, 4α,10α-dihydroxy-1α,5α*H*-guaia-6-ene (**1.2.18**) presented a discrepancy in its reported stereochemical configuration. Although Shen, et al. [35] described it as a known compound, examination of their cited reference revealed a different structure, namely guaia-6(7)-en-4α,10β-diol [35], possessing a different stereochemical configuration. Moreover, this latter compound was identified based on structural similarity to a previously reported compound, guaianediol [57]. Therefore, further investigations are required to confirm the stereochemical configuration of compound **1.2.18**.

**Figure 3:**
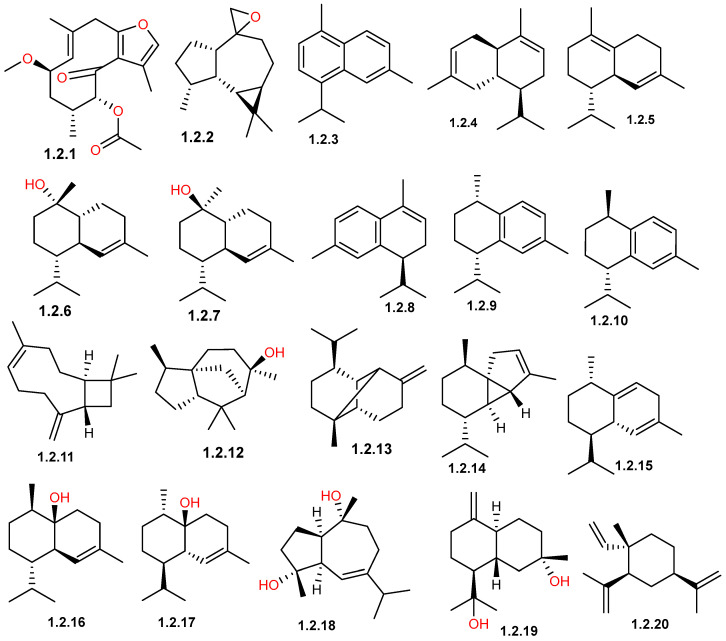
Structures of sesquiterpenes(oids) (**1.2.1**–**1.2.20**) reported from *C. gileadensis*.

**Figure 4:**
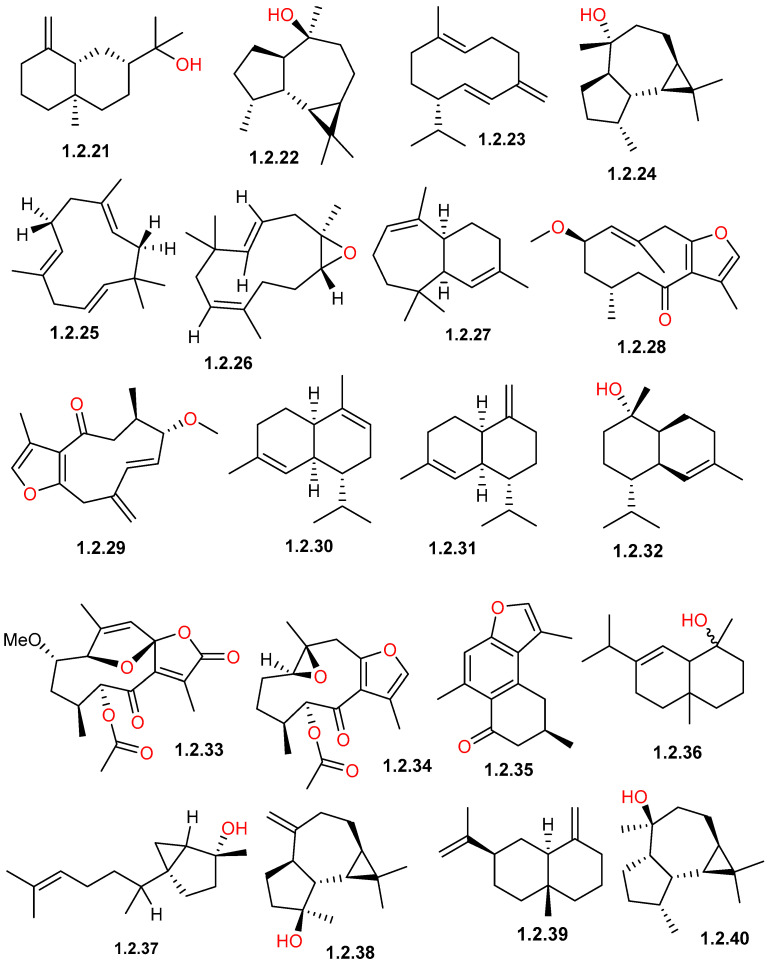
Structures of sesquiterpenes(oids) (**1.2.21–1.2.40**) reported from *C. gileadensis*.

The sesquiterpene profile of *C. gileadensis* exhibited a significant structural diversity, and its chemical composition is highly impacted by the plant part used, the preparation technique used, and the geographical origin. Members of the cadinane, germacrane, and eudesmane classes are among the principal reported compounds by many investigations (Figure 3 and Figure 4).

The distribution of sesquiterpene hydrocarbons varied considerably across different chemotypes based on variations in sources, in addition to plant parts and extraction methods. In Ein Gedi, Palestine, the leaves and fruits extracted with MTBE were dominated by β-caryophyllene (**1.2.11**) (20.12%) and germacrene D (**1.2.23**) (19.62%) [34]. However, in samples from Medina (Badr) region, the analysis of a 70% ethanolic extract from fresh aerial parts revealed a profile particularly rich in copaene (**1.2.13**) (11.48%), α-muurolene (**1.2.30**) (9.01%), and β-selinene (**1.2.39**) (5.02%) [11]. By contrast, samples from Makkah processed via hydrodistillation of fresh aerial parts and flowering tops show a prevalence of α-calacorene (**1.2.8**) (9.4%), cadalene (**1.2.3**) (5.4%), and δ-cadinene (**1.2.5**) (4.8–5.0%) [33]. Furthermore, samples from the Breiman region utilizing successive solvent extraction of shoots and aerial parts identified γ-himachalene (**1.2.27**) (21.43%) as a major constituent, highlighting the structural diversity of sesquiterpenes present within the species across different Saudi Arabian localities [32].

Another key class of the *C. gileadensis* profile is oxygenated sesquiterpenoid derivatives (Table 1 and Table 2; Figure 3 and Figure 4). In Saudi Arabian samples, viridiflorol (**1.2.40**) and spathulenol (**1.2.38**) are commonly reported as major components; in the Breiman region, viridiflorol reached 9.63%, while in Makkah and Khulais, it reached 4.9–5.41% [32,33]. Furthermore, dominant sesquiterpenoid markers in Makkah and Khulais samples have been found to be α-cadinol (**1.2.7**) (10.1%) and β-eudesmol (**1.2.21**) (11.9%) [32,33]. The chemical complexity of this secondary metabolite class was highlighted by the report of two unique isolated sesquiterpenoids, myrrhanolide D (**1.2.33**) and myrrhasin A (**1.2.34**) (Figure 4), from resin samples imported from India and extracted by refluxing with ethyl acetate [35].

#### 4.1.3. Diterpenes

Diterpenes are isoprene-derived compounds consisting of four isoprene units (C_20_). They are classified according to rings in their chemical structures into acyclic, mono-, di-, tri-, tetra-, and macrocyclic, in addition to other miscellaneous structures.

**Figure 5:**
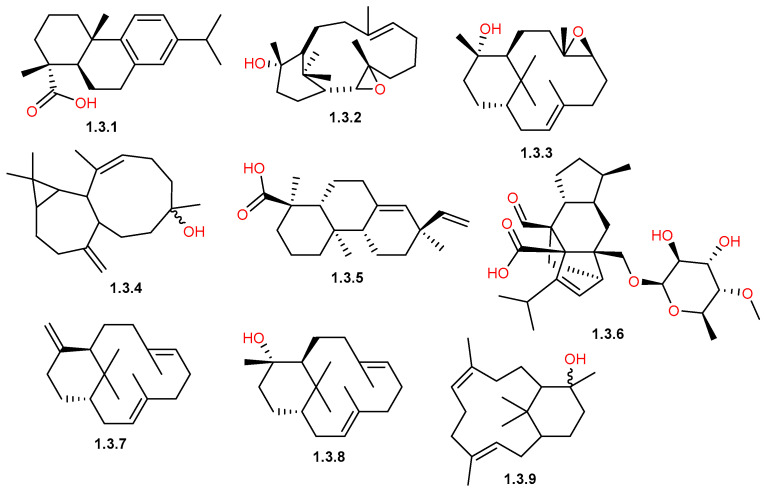
Structures of diterpenoids (**1.3.1–1.3.9**) reported from *C. gileadensis*.

The diterpenoids reported in *C. gileadensis* represent a chemically diverse group, with a significant existence of verticillane-type and resin acid derivatives (Table 1 and Figure 5). These compounds are distributed across various plant materials, including stems, leaves, bark, and resinous exudates.

Verticillanes are a predominant class of diterpenoids identified in the stems of the plant. (13*S*,14*S*)-*ent*-13,14-epoxyverticillol (**1.3.2**) and (9*S*,10*S*)-*ent*-9,10-epoxyverticillol (**1.3.3**) are two epoxy-verticillane derivatives isolated along with the alcohol derivative, *ent*-verticillol (**1.3.8**), from the stem extract of *C. gileadensis* [31,39]. Additionally, (1*S*,3*E*,7*E*,11*R*)-(+)-verticilla-3,7,12(18)-triene (**1.3.7**) was remarkable for its presence in both organic extracts and the essential oil of the stems [31,36,39].

The resin and balsam of the plant served as a primary source for diterpene acids or resin acids. Dehydroabietic acid (**1.3.1**), an abietane-type diterpenoid, was only identified in the resin of *C. gileadensis*, indicating the role of secretory tissues as major sites for diterpenoid acid accumulation. Similarly, sandaracopimaric acid (**1.3.5**), a pimarane-type diterpenoid acid, was isolated from resin or balsam, supporting the accumulation of diterpenoid acids in resinous matrices [38].

The plant leaves extracted with integrated ultrasonic-microwave-assisted methods showed the presence of the diterpenoid, sordarin (**1.3.6**). This extract displayed a capacity to synthesize silver nanoparticles with enhanced antibacterial properties [40].

#### 4.1.4. Triterpenes

Triterpenes are a diverse group of natural compounds with a 30-carbon backbone that are composed of six isoprene units and include steroids, bile acids, and saponins [58]. The majority of triterpenoids are tetracyclic (6-6-6-5; such as lanostanes, cycloartanes, dammaranes, euphanes, and tirucallanes) and pentacyclic (6-6-6-6-5 or 6-6-6-6-6; such as friedelanes, lupanes, oleananes, ursanes, hopanes, and taraxasteranes) types, although acyclic, monocyclic, bicyclic, tricyclic, and hexacyclic triterpenoids have been reported from natural sources, with pentacyclic triterpenoids representing the largest occurrence in nature [59].

**Figure 6:**
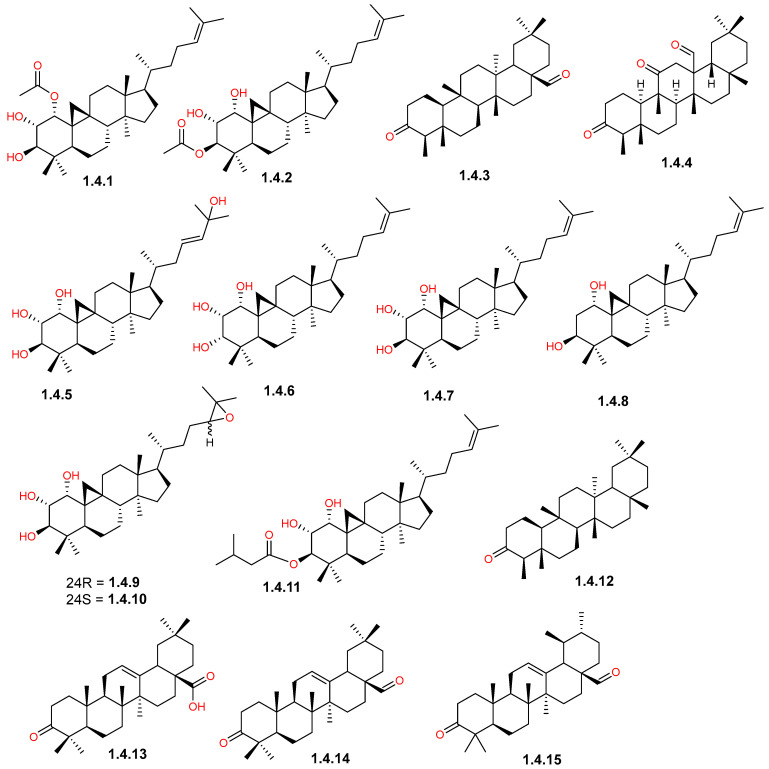
Structures of triterpenoids (**1.4.1–1.4.15**) reported from *C. gileadensis*.

The triterpenoid contents reported in *C. gileadensis* included both tetra- and pentacyclic-type derivatives, which can be categorized into the cycloartane, friedelane, oleanane, and ursane classes (Table 1). They were found to be distributed in the resin, stems, and aerial parts of the plant. Resinous exudates were rich in acetylated, hydroxylated, and epoxy derivatives of the tetracyclic cycloartane-type triterpenoids, as displayed in Figure 6. The acetoxycycloartane derivatives included 1α-acetoxycycloartan-24-ene-2α,3β-diol (**1.4.1**) and 3β-acetoxycycloartan-24-ene-1α,2α-diol (**1.4.2**), along with multiple hydroxylated derivatives, including cycloartan-23*E*-ene-1α,2α,3β,25-tetrol (**1.4.5**), cycloartan-24-ene-1α,2α,3α-triol (**1.4.6**), cycloartan-24-ene-1α,2α,3β-triol (**1.4.7**), and cycloartan-24-ene-1α,3β-diol (**1.4.8**) [41]. Two epoxycycloartane derivatives, including 24*R*,25-epoxycycloartane-1α,2α,3β-triol (**1.4.9**) and its 24*S*-epimer (**1.4.10**), as well as the valerate ester derivative of **1.4.6**, namely 3β-isovaleroyloxycycloartan-24-ene-1α,2α-diol (**1.4.11**), were also reported in the resin [41]. In contrast, the stems and aerial parts showed the presence of pentacyclic triterpenoids with friedelane, oleanane, and ursane skeletons, such as canophyllal (**1.4.3**) and friedelin (**1.4.12**), oleanonic acid (**1.4.13**) and oleanonic aldehyde (**1.4.14**), and the ursane-type urs-12-en-3-one-28-al (**1.4.15**) [39,42]. Remarkably, the stems showed the presence of the friedelan-type triterpenoid, commigileadin A (**1.4.4**) [39,43].

### 4.2. Phytosterols

**Figure 7:**
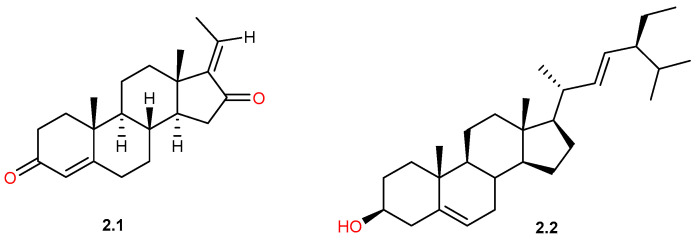
Structures of phytosterols (**2.1–2.2**) reported from *C. gileadensis*.

Phytosterols are triterpene-derived metabolites, but undergo structural rearrangement and side-chain modification, resulting mostly in the production of C28 (e.g., campesterol) and C29 (e.g., β-sitosterol, stigmasterol) sterols. They are characterized by the presence of a sterane (steroid) ring, a β-hydroxyl group at C-3, and unique alkyl side chains at the C-24 position and serve as structural components of plant cell membranes [60]. Several studies reported the existence of phytosterols in the aerial parts, leaves, and resin of *C. gileadensis*, including guggulsterone (**2.1**) and stigmasterol (**2.2**), Figure 7 [35,43,44].

### 4.3. Flavonoids

Flavonoids are a major subclass of polyphenols, distinguished by a C6-C3-C6 structural framework, consisting of two aromatic rings linked by a three-carbon bridge [61,62]. In the plant kingdom, these secondary metabolites are essential for pigmentation, UV protection, and defense against environmental stressors [63].

**Figure 8:**
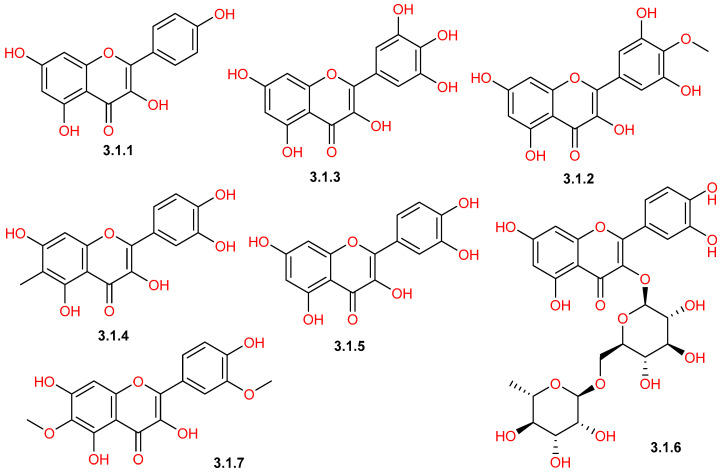
Structures of flavonols and their derivatives (**3.1.1–3.1.7**) reported from *C. gileadensis*.

**Figure 9:**
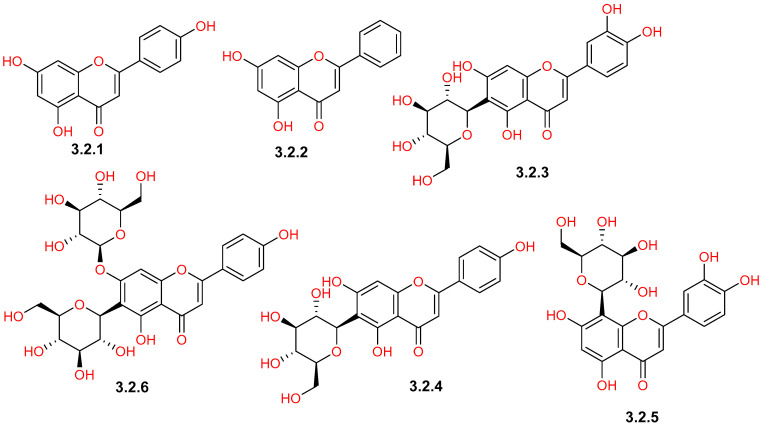
Structures of reported flavones and their derivatives (**3.2.1–3.2.5**) from *C. gileadensis*.

**Figure 10:**
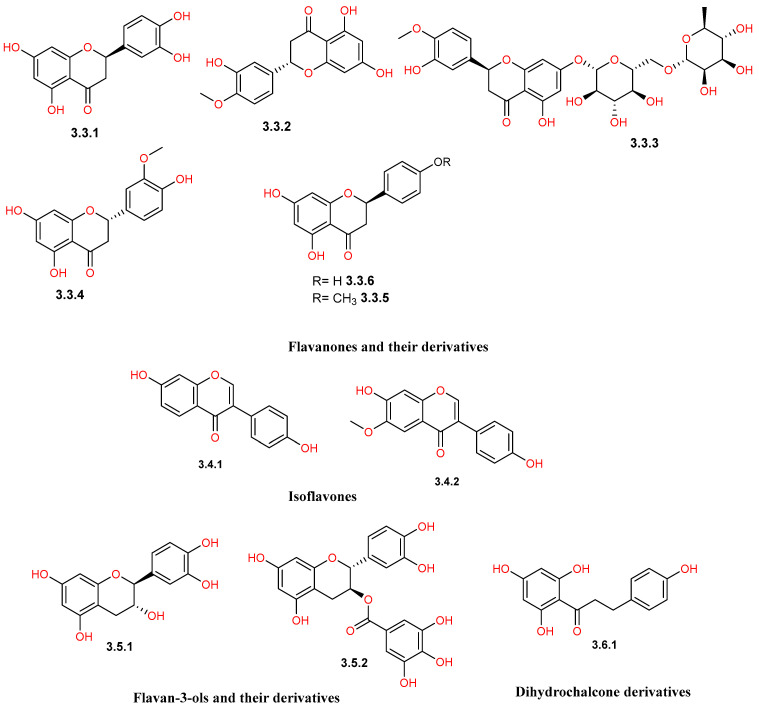
Structures of reported flavanones (**3.3.1–3.3.6**), isoflavones (**3.4.1–3.4.2**), flavan-3-ols (**3.5.1–3.5.2**), and chalcone (**3.6.1**) derivatives from *C. gileadensis*.

**Figure 11:**
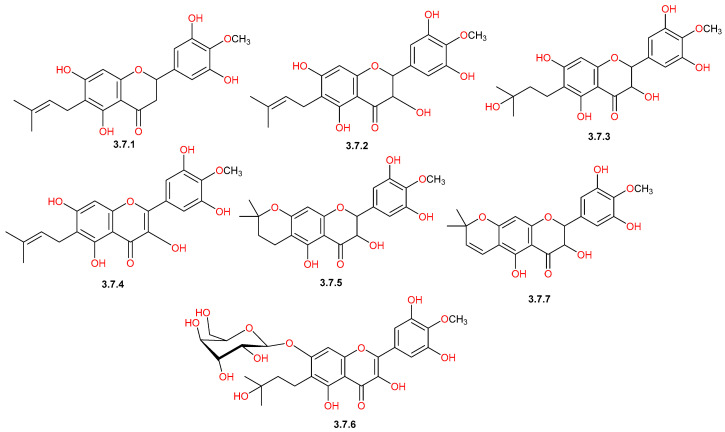
Structures of prenylated flavonoids and their derivatives (**3.7.1–3.7.7**) reported from *C. gileadensis*.

Reported phytochemical studies on *C. gileadensis* revealed a rich flavonoid profile of *C. gileadensis*, demonstrating a wide spectrum of structural diversity (Table 1 and Figure 8, Figure 9, Figure 10 and Figure 11). Flavonols represented one of the major classes (Figure 8), comprising seven reported compounds with quercetin (**3.1.5**) as the most commonly detected flavonol in different plant materials, including aerial parts, leaves, and bark [25,32,40,42,43,45]. This class also included kaempferol (**3.1.1**) and mearnsetin (**3.1.2**), which are mainly extracted from aerial parts and bark [42,43,45,46]. Within the flavones class (Figure 9), apigenin (**3.2.1**) was isolated from both aerial parts and bark [11,45], whereas saponarin (**3.2.6**) was only detected in leaf extracts [40]. The flavanone group (Figure 10) was represented by compounds, such as naringenin (**3.3.6**), which was detected in both aerial parts and bark [11,43,45], and hesperidin (**3.3.3**), detected in the aerial parts [11]. Furthermore, the plant is characterized by the presence of a unique prenylated flavonoid group (Figure 11), such as comophorin A (**3.7.1**) and comophorin B (**3.7.2**), which are specifically reported in the stem bark [45,46,47]. Finally, other minor classes contributed to this flavonoid profile, including isoflavones such as daidzein (**3.4.1**) and dihydrochalcones such as phloretin (**3.6.1**), both of which were identified in bark extracts, as displayed in Figure 10 [45].

### 4.4. Phenolic Acids

Phenolic acids are a significant subclass of plant polyphenols characterized by a phenol moiety and a resonance-stabilized structure, which enables free radical scavenging through hydrogen atom donation [61,63]. These compounds are generally subdivided into two main subgroups, including hydroxybenzoic acids and hydroxycinnamic acids, with the latter subgroup comprising caffeic and ferulic acids, which are commonly found in edible plants [62].

**Figure 12:**
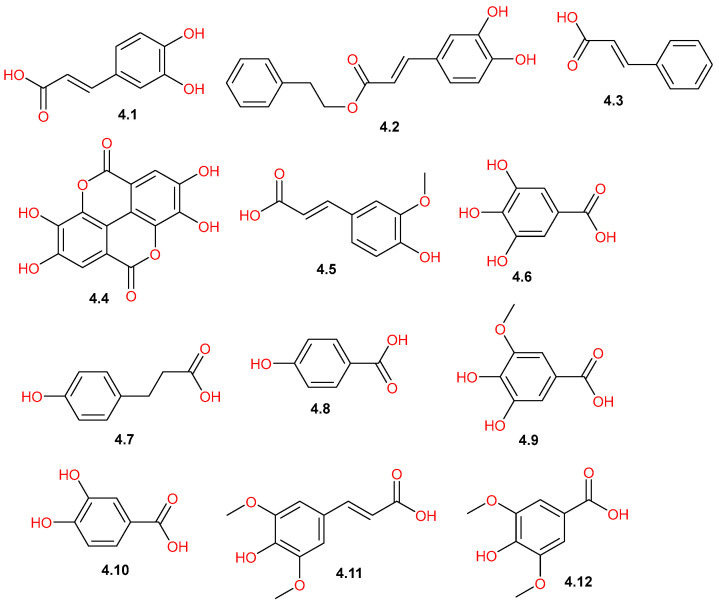
Phenolic acid derivatives (**4.1–4.12**) reported from *C. gileadensis*.

Reported phytochemical investigations of *C. gileadensis* identified a diverse group of phenolic acids (Figure 12) found particularly within the stem bark [45]. The hydroxycinnamic acid subgroup was represented by compounds such as caffeic acid (**4.1**), cinnamic acid (**4.3**), ferulic acid (**4.5**), hydro-*p*-coumaric acid (**4.7**), and sinapic acid (**4.11**), which were detected in the bark extract [45], while caffeic acid phenethyl ester (**4.2**) was identified in the aerial parts [11]. On the other hand, the hydroxybenzoic acid subgroup comprised gallic acid (**4.6**), which appeared to be abundant in the aerial parts and the bark [11,45]. Additionally, several benzoic derivatives were detected in the bark, including 4-hydroxybenzoic acid (**4.8**), 3-*O*-methyl gallic acid (**4.9**), and protocatechuic acid (**4.10**) [45]. Notably, syringic acid (**4.12**) was isolated from aerial part extract [42], while ellagic acid (**4.4**) was detected in the bark extract [45].

### 4.5. Lignans

Lignans are a large group of polyphenols that function as structural building blocks of plant cell walls. These compounds are typically dimers, or occasionally trimers and tetramers, of phenylpropane units (C6-C3), known as monolignols, formed through oxidative coupling reactions [64].

**Figure 13:**
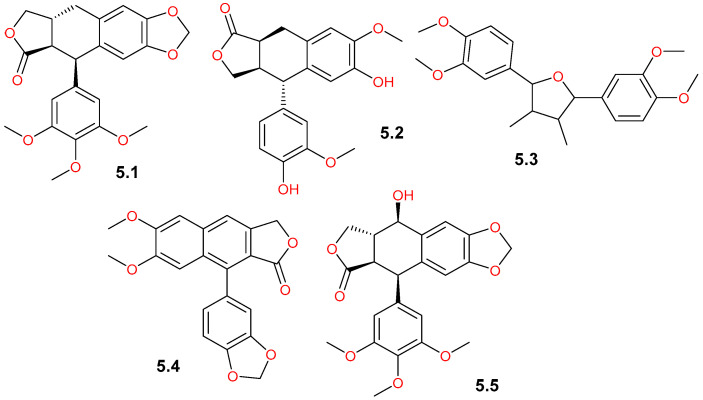
Lignan derivatives (**5.1–5.5**) reported from *C. gileadensis*.

By reviewing the available literature, lignans were found to be identified in the leaves of *C. gileadensis*. Several lignan derivatives (Table 1 and Figure 13) were tentatively identified by UPLC-qTOF-MS in the leaf extracts, including anthricin (**5.1**), *β*-conidendrin (**5.2**), (-)-galbelgin (**5.3**), and the medicinally important lignan, podophyllotoxin (**5.5**) [40]. Furthermore, a biotechnological study utilizing tissue culture has tentatively identified justicidin B (**5.4**) in the leaves, callus, and cell suspension cultures of *C. gileadensis* by LC-MS/MS analysis, suggesting that the phenylpropanoid pathway remains active in undifferentiated cell systems [23].

### 4.6. Coumarins

Coumarins are a class of naturally occurring benzopyrone compounds composed of a benzene ring fused to a pyrone ring. These natural products are aromatic and fragrant; their aroma often resembles woodruff, fresh hay, or vanilla. Beyond their organoleptic characteristics, coumarins demonstrate a wide spectrum of biological activities, which are dependent on their chemical substitutions and structural configurations [65].

**Figure 14:**
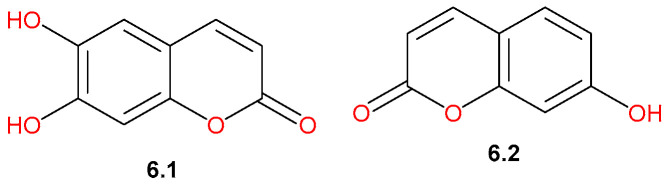
Coumarins (**6.1–6.2**), reported from *C. gileadensis*.

Reported phytochemical studies targeting analysis of the bark extract of *C. gileadensis* using the LC-MS/MS technique have led to the tentative identification of two coumarin derivatives (Figure 14), including 6,7-dihydroxycoumarin (**6.1**) and umbelliferone (**6.2**) [45].

### 4.7. Fatty Acids and Lipids

Lipids are oily, fatty, or waxy compounds soluble in organic solvents, comprising four primary subgroups: fats and oils (triacylglycerols), phospholipids, waxes, and steroids [66]. Fatty acids serve as the fundamental building blocks for these lipids and are characterized as hydrophobic organic molecules with unique aliphatic chains, either saturated or unsaturated, terminating in a carboxylic acid moiety, commonly 12 to 18 carbons in length [67]. The chain length, branching, and degree of saturation determine the physical properties of lipids and their biological role [66,67].

**Figure 15:**
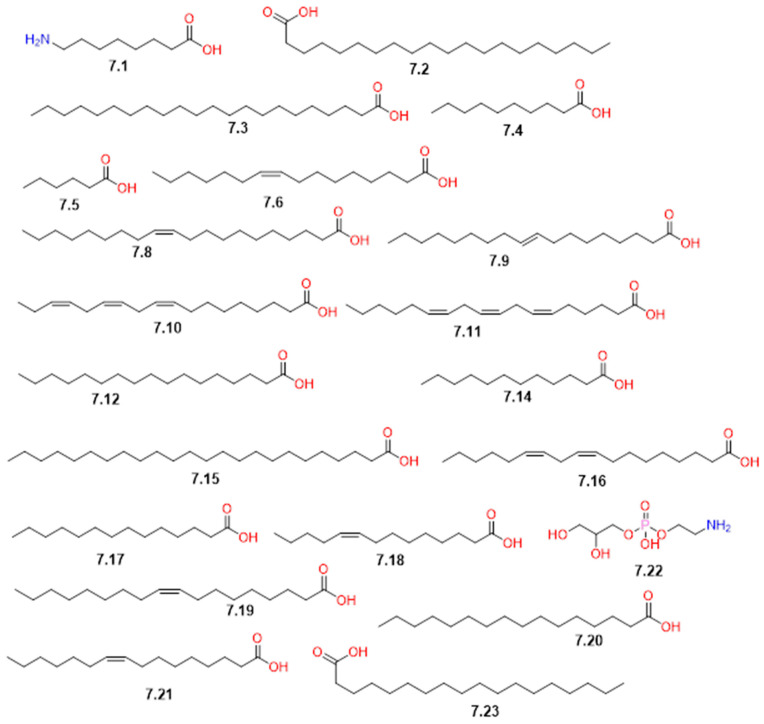
Fatty acid derivatives (**7.1–7.23**), excluding the tentatively identified lipids (**7.7** and **7.13**) reported from *C. gileadensis*.

In *C. gileadensis*, this class is represented by a variety of derivatives identified in various plant parts (Table 1 and Figure 15). This class can be categorized into four distinct groups with various lengths, substitutions, and degrees of saturation: saturated fatty acids, unsaturated fatty acids, fatty acid derivatives, and special lipids. Saturated fatty acids constituted the most abundant class detected in the resin, bark, and stem. Among these, palmitic acid (**7.20**), caprylic acid (**7.6**), and stearic acid (**7.23**) were found in high concentrations within the resinous balsam at 15.50%, 12.60%, and 10.73%, respectively [48]. While palmitic acid (**7.20**) is distributed across the stem and resin [37,48], stearic acid (**7.23**) and arachidic acid (**7.2**) were found in both the bark and resin [45,48]. This group also included behenic (**7.3**), capric (**7.4**), caproic (**7.5**), heptadecanoic (**7.12**), lauric (**7.14**), lignoceric (**7.15**), and myristic (**7.17**) acids, which were reported mainly in the resinous balsam [48].

On the other hand, the unsaturated fatty acids, oleic acid (**7.19**) was the most abundant member of this group, comprising 33.36% of the balsam oil [48]. This class also comprised monounsaturated acids, such as palmitoleic (**7.21**) detected in the bark and resin [45,48], alongside myristoleic (**7.18**), elaidic (**7.9**), and *cis*-11-eicosenoic (**7.8**) acids in the resin [48]. Polyunsaturated fatty acids, including linoleic (**7.16**), γ-linolenic (**7.11**), and α-linolenic (**7.10**) acids, have also been reported in the resin [48]. Regarding fatty acid derivatives, the amino-fatty acid 8-aminocaprylic acid (**7.1**) was exclusively identified in leaf extracts [40]. Finally, special lipids were tentatively identified through LC-MS analysis in the aerial parts of *C. gileadensis*, including leaves and branches. They included three compounds: ceramide (**7.7**), hexosylceramide (**7.13**), and phosphatidylethanolamine (**7.22**) [49].

### 4.8. Alkaloids

**Figure 16:**
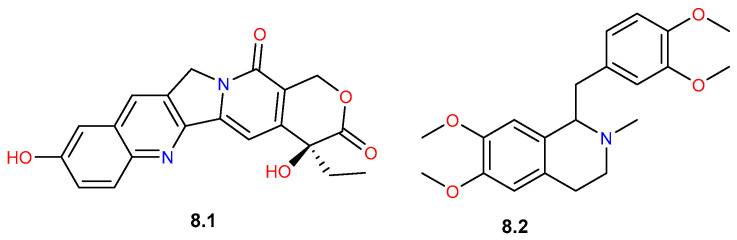
Alkaloids (**8.1–8.2**) reported from *C. gileadensis*.

Alkaloids class is an unusual family in the *Commiphora* genus. However, two alkaloids have been tentatively determined during LC-MS/MS-based chemical profiling of the plant and its tissue culture (Figure 16) [23]. 10-Hydroxycamptothecin (**8.1**) was detected only in the methanol extract of the leaves, whereas laudanosine (**8.2**) was detected in cell suspension culture, as well as in the seeds and leaves of the wild plant [23].

### 4.9. Miscellaneous Compounds

**Figure 17:**
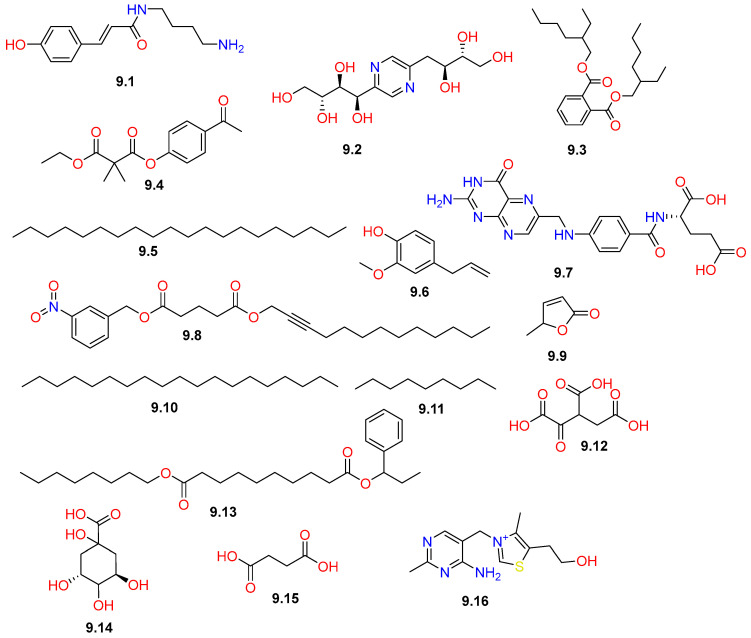
Miscellaneous group (**9.1–9.16**) reported from *C. gileadensis*.

The miscellaneous group of compounds (**9.1–9.16**) displayed in Figure 17 comprises a diverse array of bioactive metabolites identified in various plant materials of *C. gileadensis*, including the bark, leaves, stems, and aerial parts (Table 1).

They represented several chemical classes, including vitamins, organic acids, nitrogenated compounds, and long-chain hydrocarbons (Figure 17). Particularly, the bark showed the presence of nitrogen-containing derivatives, including a phenolamide, coumaroylputrescine (**9.1**), and a pyrazine derivative, 2,6-deoxyfructosazine (**9.2**), as well as carboxylic acids, including oxydisuccinic acid (**9.12**), quinic acid (**9.14**), and succinic acid (**9.15**) [45]. Investigations of the leaves revealed the presence of several miscellaneous esters, including dimethylmalonic acid, 4-acetylphenyl ethyl ester (**9.4**), glutaric acid, tridec-2-yn-1-yl 3-nitrobenzyl ester (**9.8**), and sebacic acid, octyl 1-phenylpropyl ester (**9.13**) [40]. The aerial parts were noted for their di-(2-ethylhexyl)-phthalate (**9.3**) content, in addition to aliphatic hydrocarbons, including eicosane (**9.5**), and nonadecane (**9.9**) [32], as well as nonane (**9.11**). It is worth noting that the presence of the phthalate derivative (**9.3**) in the aerial part extract most likely arises from contamination of the extraction solvents rather than being an endogenous plant constituent, as phthalate esters are well-documented contaminants introduced during solvent extraction and analytical procedures [68]. Additionally, eugenol (**9.6**) was identified in both the aerial parts and stems [33]. Finally, the stem was found to be a source of essential nutrients, such as folic acid (**9.7**), 5-methyl-2(5)-furanone (**9.9**), and vitamin B1 (**9.16**) [37].

### 4.10. Phytochemistry of Essential Oil

The aromatic and bioactive nature of the plant is highly attributed to the abundant terpenoid content found in the essential oil. Research on the essential oil (EO) of *C. gileadensis* revealed distinct chemotypic divergence, with its composition significantly based on geographical origin, the plant organ processed, and the specific extraction or analytical techniques used [11,20,32,33]. Table 2 summarizes these variation factors and compares the major chemical components identified by different studies.

The comparative data presented in Table 2 highlighted marked variability in the EO composition of *C. gileadensis*, which can be directly attributed to differences in geographical origin, plant organ, and extraction or preparation methods. EOs obtained from the Makkah region, Saudi Arabia, were consistently dominated by sesquiterpenes; for example, hydrodistilled oils from fresh aerial parts contained α-calacorene (9.4%), terpinen-4-ol (8.5%), and δ-cadinene (5.0%), while oils from stored aerial material showed the presence of more stable oxygenated sesquiterpenes, such as α-cadinol (10.1%), spathulenol (5.8%), and viridiflorol (4.9%) [33]. In contrast, samples collected from the Badr area, Medina, showed an obviously different chemotype, with steam-distilled oils rich in monoterpene hydrocarbons, particularly β-myrcene (17.44%) and β-phellandrene (9.59%), alongside nonane (10.88%) and the diterpene verticiol (10.56%) [11]. These findings support the influence of geographical variation and local environmental conditions on terpene biosynthesis.

Organ-based variation was also evident. Fresh flowering tops from Makkah were rich in terpinen-4-ol (9.8%), whereas fresh aerial parts from the same region were dominated by α-calacorene (9.4%) [33]. EOs extracted from leaves and fruits cultivated in Ein Gedi (Palestine) exhibited a balanced profile of sabinene (21.11%), β-caryophyllene (20.12%), and germacrene D (19.62%), reflecting mixed mono- and sesquiterpene biosynthesis [34]. The resin and exudate represented a distinct chemical niche. It was mainly made up of monoterpene hydrocarbons. Direct injection analysis showed sabinene at 43.8% and α-pinene at 24.0%. HS-SPME results highlighted sabinene at 46.4% and α-pinene at 25.8%. It also detected other highly volatile components, including α-thujene at 4.3%.

The extraction technique exerts a crucial effect on the chemical profile of the produced EO. Steam or hydrodistillation methods preferentially recover volatile monoterpenes, while solvent-based methods selectively enrich heavier sesquiterpene hydrocarbons. This was illustrated in Medina samples, where steam-distilled oil was characterized by 36.9% monoterpene hydrocarbons dominated by β-myrcene, while a 70% ethanolic extract of the same material was rich in sesquiterpenes, including copaene (**1.2.13**) (11.48%), α-muurolene (**1.2.30**) (9.01%), and β-selinene (**1.2.39**) (5.02%) [11].

Taken together, the pronounced chemical variability of EOs from *C. gileadensis* emphasizes the need for a standardized source and methodology that will allow a valid comparison of chemical composition and biological activity.

**Figure 18:**
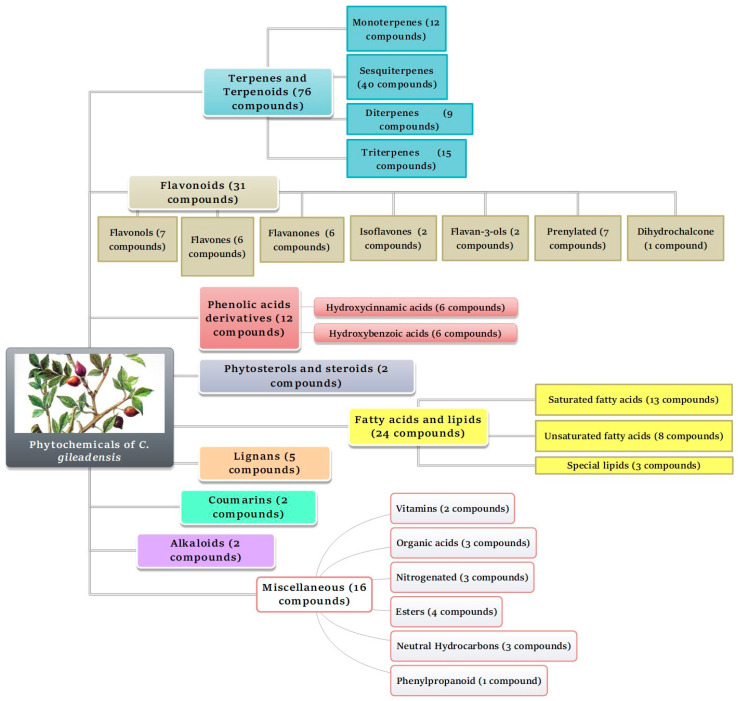
A summary of the identified phytochemical groups in *C. gileadensis*.

The phytochemical classes reviewed in this study highlight the remarkable chemical diversity of *C. gileadensis* (Figure 18).

## 5. Ethnobotanical and Traditional Uses of *C. gileadensis*

The title plant, *C. gileadensis* has a long history of use in traditional medicine, particularly in ancient and Arabic medicine (Table 3). It was recognized by names such as *Arabian balsam*, Bisham, becham, or balessan medicine [23,44,69]. Historically, the sap or resin was the most highly valued material, which was utilized topically for thousands of years as a potent antiseptic and wound-healing agent, particularly in skin conditions such as burns, wounds, and skin infections [23,44,49,69]. Beyond topical applications, the resin was traditionally indicated for headaches, paralysis, hearing disorders, stroke, respiratory ailments, fractures, gastrointestinal ailments, arthritis, and weight reduction, as well as an antidote for scorpion stings and snake bites. It was even applied to the eyes to treat conditions such as cataracts and blurred vision [11,49,69]. The plant bark, especially the inner bark, was frequently employed as an antiseptic for injuries and, when ground into juice, as an anti-allergic treatment for skin conditions, such as burns, eczema, or inflammation [33,37,70]. Moreover, the bark aqueous extract was used as an anti-hypertensive [71]. Traditionally, local populations in Palestine and other Arab regions have utilized the decoction of the flowers and leaves to manage pain, constipation, and to promote urine output or to expel renal calculi [13,71,72,73,74]. Crushed leaves were used in the treatment of eye tumors [17]. Medicinal preparations from seeds and wood were utilized for chest, kidney, and stomach complaints, and to relieve conditions, including rheumatism, scurvy, and jaundice [69,72]. In traditional gynecology, particularly in Yemen, various plant organs have been used to mitigate labor pain, as a contraceptive, and to treat cervical infections [37,69,73]. In Syria, the oil imported from Egypt was particularly indicated for cold, ear problems, excess phlegm (catarrh), and to massage arthritic joints [69]. Other traditional uses of the oil included treatment of epilepsy, tetanus, and gonorrhea [40,69]. Additionally, the twigs were utilized as a natural toothbrush (miswak) to maintain oral hygiene and were traditionally boiled to extract oil for treating traumatic injuries [33,37,69]. In certain regions, such as Oman, extracts were even documented as a historical treatment for rabies and as a cleansing bath for newborns [37].

## 6. Pharmacological Activities Reported for *C. gileadensis*

A wide pharmacological potential was reported for *C. gileadensis*, which was supported by its rich and varied secondary metabolite profile, Table 1 (such as terpenoids and polyphenols), validating its historical and current medicinal applications. Numerous studies were conducted using various in vitro, in vivo, or ex vivo models to investigate multiple bioactivities of various plant parts and unorganized products, including the resin, essential oil, polysaccharide, or crude extracts of *C. gileadensis* prepared by diverse extraction techniques, as well as drying conditions, and derived from different localities. Additionally, the traditional medicinal uses of the plant have been scientifically validated through numerous reported studies. Some reported biological activities for *C. gileadensis* are summarized in Table 4.

### 6.1. Antioxidant Activity

An imbalance between the generation of free radicals and the ability of cells to neutralize or eradicate them leads eventually to oxidative stress. It contributes to the development of many chronic and degenerative conditions, such as cancers, Alzheimer’s disease, and cardiovascular problems. Oxidative stress may result from a number of predisposing factors, such as smoking, UV radiation, and environmental irritants [93]. Antioxidants are molecules that have the capacity to neutralize free radicals, preventing their interaction with vital macromolecules, such as lipids, proteins, and DNA. Therefore, they reduce processes that can contribute to chronic and degenerative conditions through inhibiting lipid peroxidation, protein oxidation, and oxidative DNA damage [94].

Plant polyphenols and terpenoids are antioxidant molecules that exert their activity through several mechanisms, including scavenging and neutralization of free radicals, chelation of metals that catalyze the generation of free radicals (such as iron, copper, zinc, cadmium), and stimulating the secretion of antioxidant enzymes, such as superoxide dismutase (SOD), catalase (CAT), glutathione peroxidase (GPx). Furthermore, they impede the activity of enzymes, which produce reactive oxygen species (ROS), such as NADPH oxidase and xanthine oxidase, and restore the activity of other functioning antioxidants, such as vitamin C and vitamin E [95,96,97].

The antioxidant potential of various plant parts of *C. gileadensis* has been extensively studied, displaying significant activities in both in vitro and in vivo models. In the in vitro models, the extracts investigated demonstrate potent radical scavenging activity using the DPPH, ABTS, and H_2_O_2_ assays. Reported studies comparing the antioxidant potential of the extracts of plant parts indicated that stem peel extracts were more potent radical scavengers than leaf extracts, with reported EC_50_ values of 1.06 µg/mL for the stem extract compared to 3.39 µg/mL for leaf extract by the DPPH method [80]. Extraction procedure significantly impacted these results; for instance, ultrasonic-assisted extraction (USE) of leaves yielded a higher total phenolic content (TPC) than the hydrodistillation method (118.71 vs. 101.47 mg GAE/g DM) and also exhibited greater DPPH radical scavenging [78]. Furthermore, the steam-distilled EO showed stronger radical scavenging activity (IC_50_ 22.2 µg/mL) compared to 70% ethanolic extracts (IC_50_ 56.5 µg/mL); however, the ethanolic extract was notably effective in the β-carotene bleaching (BCB) assay (IC_50_ 75.8 µg/mL) [11].

The in vivo models using diabetic rats demonstrated that *C. gileadensis* extracts effectively mitigated oxidative stress by strengthening the natural body defense. The aqueous extracts from twigs and leaves were shown to induce the activities of CAT, SOD, and GST, while reducing levels of malondialdehyde (MDA), a marker of lipid peroxidation [79]. Similarly, the butanol fraction of stem bark at a dose of 100 mg/kg has been shown to increase reduced glutathione (GSH) by 82.51% and SOD by 49.03%, while reducing MDA by 33.41% compared to the diabetic control group [45]. The methanol extract of the stem also exhibited high ferric reducing antioxidant power (FRAP) (1.95 mM FE/mg), which was comparable to the reference drug, ascorbic acid [81].

The reported antioxidant potential was largely attributed to the high concentration of terpenoids, polyphenols, and flavonoids. Monoterpenes, such as α-pinene (**1.1.5**), β-pinene (**1.1.6**), sabinene (**1.1.8**), γ-terpinene (**1.1.9**), and terpinen-4-ol (**1.1.10**), were suggested as major contributors to the free radical scavenging activity [11,78]. Similarly, sesquiterpenes, including β-caryophyllene (**1.2.11**), β-copaene (**1.2.13**), and germacrene D (**1.2.23**), were related to the protective antioxidant [34,78]. Whereas phenolics and flavonoids, such as quercetin (**3.1.5**), gallic acid (**4.6**), caffeic acid (**4.1**), and rutin (**3.1.6**), were suggested to play key roles in scavenging free radicals [11].

### 6.2. Anti-Inflammatory Activity

The reported anti-inflammatory activity of *C. gileadensis* was demonstrated utilizing various experimental in vitro and in vivo models, showing significant potential in both acute and chronic inflammation (Table 4). These activities are characterized by the multi-targeted suppression of diverse biochemical mediators, immune cells modulation, and inhibition of enzymes associated with inflammatory responses. Additionally, specific preparations of *C. gileadensis* demonstrated significant effectiveness in suppressing acute and chronic inflammation at non-toxic dosage levels (Table 4).

A methanolic extract of the aerial parts (at a dose of 500 mg/kg) has been shown to significantly reduce the accumulation of Prostaglandin E2 (PGE2) (40.8%), Nitric Oxide (NO) (55.47%), and Tumour Necrosis Factor-alpha (TNF-α) (19.06%) at the site of inflammation [83]. Furthermore, the 70% ethanolic extract of the aerial parts exhibited COX-1 inhibitory activity (450 µg/mL) and prevented protein denaturation (110.5 µg/mL), a key marker for anti-arthritic potential [11]. The ethanol extracts of the aerial parts also significantly reduced carrageenan-induced paw edema and suppressed granuloma formation in rat models, indicating significant efficacy in both acute and chronic inflammatory conditions [76,82]. The aerial parts extract was demonstrated to synergistically potentiate the effects of conventional NSAIDs (non-steroidal anti-inflammatory drugs), such as diclofenac [82].

Recent studies highlighted the ability of *C. gileadensis* to regulate the immune system by altering T-lymphocyte cells. This effect was primarily evaluated using the sap, methanol, and acetone extracts of the aerial parts. The investigated extracts significantly decreased total pro-inflammatory lymphocytes and peripheral cluster of differentiation (CD) subsets, including CD3^+^, CD4^+^, and CD8^+^ [86,87]. Conversely, the extract of the aerial parts induced a significant increase in T-regulatory (Treg) cells (CD4^+^ CD25^+^ and CD8^+^ CD25^+^), which have the advantage of maintaining immune tolerance and protecting against chronic inflammatory damage [86].

The anti-inflammatory activities are mainly attributed to the rich terpenoids, steroids, and phenolic content of the plant (Table 1). Of these, key identified constituents were suggested as major contributors to the anti-inflammatory action of the plant, including the steroidal guggulsterone (**2.1**), which was isolated from the leaves, acting as a potent anti-inflammatory agent by antagonizing the bile acid receptor and suppressing the NF-κB pathway, thereby downregulating pro-inflammatory cytokines and chemokines [44,98]. Sesquiterpenes, such as β-caryophyllene (**1.2.11**), β-copaene (**1.2.13**), and germacrene D (**1.2.23**), were suggested to have anti-inflammatory actions [34,78]. The diterpeneoids (resin acids), including dehydroabietic acid (**1.3.1**) and sandaracopimaric acid (**1.3.5**), isolated from the resin, exhibited vasorelaxant and anti-inflammatory-related actions on pulmonary arteries via the PI3K/Akt-eNOS signaling pathway [38]. Finally, phenolic compounds, including caffeic acid, gallic acid (**4.6**), phenethyl ester (**4.2**), and flavonoids (Table 1) were suggested as potential contributors to the observed COX-1 inhibitory activity and protein denaturation suppression of the investigated aerial parts extract [11].

### 6.3. Anticancer Activity

The ability of extracts or certain isolated compounds of *C. gileadensis* to target malignant cells has been extensively investigated (Table 4). They demonstrated broad selective cytotoxic effects against various cancer types (Table 4).

The stem ethanol extract and EO were shown to have strong cytotoxicity against mouse lymphoma (BS-24-1) and MoFir (Epstein–Barr virus (EBV)-transformed human B lymphocytes) [34]. The extracts showed no inhibitory effect on normal human skin fibroblasts, indicating selective cytotoxicity [13,34]. The mechanism of cell death was shown to be associated with caspase-3 activation and DNA fragmentation [34]. Furthermore, the cytotoxicity of the sap was found to be cell cycle-dependent, targeting transformed cells in the S or G_2_/M phases of division, leaving cells in the pre-replicative G_1_ phase unaffected [13].

Diverse extracts and products derived from *C. gileadensis* exhibited varying degrees of potency against different cancer types. For instance, polysaccharides extracted from the stems showed potent antiproliferative activity (IC_50_ 13.15 µg/mL) against the colorectal cancer lines, SW480 and SW620, through induction of cell death via apoptosis. However, the bark extract demonstrated moderate activity (IC_50_ 24.5 µg/mL) against lung (A549) and cervical (HELA) carcinomas, while callus and cell suspension cultures derived from the plant showed a more specific activity against A549 lung carcinoma [23,85]. Furthermore, prenylated flavonoids isolated from the stem bark showed high inhibitory activity against MCF-7 (breast) and HepG2 (liver) cancer lines, with comophoroside A (**3.7.6**) showing the greatest activity (IC_50_ 8 µg/mL) [46]. The leaf extract was also reported to significantly decrease HepG2 viability at concentrations of 100 µg/mL [78].

The anticancer potential of the plant was attributed to a diverse group of phytochemicals, particularly terpenoids, flavonoids, and lignans (Table 1). In the EO, β-caryophyllene (**1.2.11**) served as a primary bioactive constituent, which constituted about 20% of the EO composition. It was shown to act as a selective inducer of caspase-3-dependent apoptosis in mouse lymphoma and human B-lymphocyte cell lines without affecting normal human skin [13,34]. Furthermore, comprehensive metabolite profiling of the plant suggested a group of identified lignans and alkaloids with established antineoplastic properties as contributors to cytotoxic properties, including 10-hydroxycamptothecin (**8.1**), justicidin B (**5.4**), and anthricin (deoxypodophyllotoxin) (**5.1**), detected in leaf, bark, and callus extracts [23,85]. Moreover, the cytotoxic potential was further supported by the flavonoid content, particularly flavonols such as quercetin (**3.1.5**) and myricetin (**3.1.3**), as well as prenylated flavonoids isolated from the stem bark [23,46]. Notably, the prenylated flavonoid comophoroside A (**3.7.6**) demonstrated potent inhibitory action against MCF-7 and HepG2 cell lines [46].

### 6.4. Antidiabetic Activity

Research on *C. gileadensis* revealed hypoglycemic potential, insulin level restoration, and immune system modulation to preserve pancreatic integrity. Numerous experimental models have confirmed these effects (Table 4). The leaves, twigs, and stem bark extracts demonstrated a potent hypoglycemic effect. In streptozotocin (STZ)-induced diabetic models, the butanol fraction of the stem bark significantly reduced blood glucose and α-amylase levels and increased insulin levels [45]. Similarly, the aqueous extracts of twigs and leaves were shown to significantly decrease fasting blood sugar and glycated hemoglobin (HbA1c) in alloxan-induced diabetic rats, with the twigs extract exhibiting higher potency than the leaf extract [79]. Evaluations of the antidiabetic properties of the sap and acetone extracts of the aerial parts in STZ-induced diabetic mice revealed that the sap-treated group achieved normal blood glucose levels after only 6 days of treatment, while the acetone group required 15 days [87].

The antidiabetic properties of the plant were highly attributed to its antioxidant capacity and ability to modulate inflammatory responses associated with diabetes. It enhances endogenous antioxidant defenses, such as SOD and GSH, while reducing lipid peroxidation markers, thereby protecting pancreatic β-cells from oxidative damage [45,79]. They also included inhibition of α-amylase, reducing the breakdown of carbohydrates into glucose [45]. Histologically, it was reported that the group of diabetic rats treated with *C. gileadensis* twig aqueous extract restored normal pancreatic tissues compared to the pancreatic tissues in the untreated group, which showed vacuolation of Langerhans’ islets [79]. Immunological parameters such as IgA, IgG, and IgM were also associated with diabetes. Diabetic patients may have altered levels of IgA, IgG, and IgM, which can affect their immune response, making them more susceptible to infections. Twigs aqueous extract was found to be effective in restoring the immunoglobulin levels [79]. Sap and aerial parts extracts were shown to reduce pro-inflammatory CD4^+^ and CD8^+^ T-lymphocyte subsets in diabetic models. Conversely, they increased the population of Treg cells, CD4^+^ CD25^+^ and CD8^+^ CD25^+^, which help maintain immune tolerance and may prevent the progressive autoimmune destruction of pancreatic β-cells [86,87].

Phytochemical studies identified several potentially bioactive metabolites that may be responsible for these antidiabetic activities. These included 2,6-deoxyfructosazine (**9.2**) and saponarin (**3.2.6**), which were identified in the leaf extract and are known to regulate blood sugar levels [40]. Other potential bioactive compounds included phenolics and flavonoids, quercetin (**3.1.5**), gallic acid (**4.6**), caffeic acid (**4.1**), and naringenin (**3.3.6**), which were identified in the bioactive fractions and are linked to α-amylase inhibition and antioxidant protection [45]. In addition to the previously mentioned antioxidant compounds, which are believed to play a role in mitigating oxidative stress, these compounds may also attenuate the induced progression of diabetes [34,78].

### 6.5. Wound Healing Activity

The wound healing process is a physiological response initiated after skin injury, involving successive and overlapping functions, including haemostasis (stopping bleeding), inflammation, cell proliferation and maturation, and remodeling, mediated through several cytokines, chemical mediators, and secretions from different types of cells [99]. Studies of the wound healing properties of *C. gileadensis* (Table 4) showed that it accelerated tissue regeneration, exhibited potent antimicrobial action at the injury site, and exerted significant anti-inflammatory modulation [36,49]. Topical application of the aerial parts (leaves, branches, fresh stems, and EO) extract significantly enhanced the rate of wound reduction and shortened the epithelialization period in both infected (*S. aureus*) and non-infected excision models [36,49]. Histopathological evaluations demonstrated that the extracts reduced inflammatory cell infiltration, promoted earlier collagen fiber deposition, and stimulated rapid re-epithelization and granulation tissue formation [36,49]. These effects were attributed to a high ceramide content of (69% of methanolic extract), which was able to restore skin barrier function. Additionally, the wound healing potential was supported by the terpenoid compounds such as β-caryophyllene (**1.2.11**), β-pinene (**1.1.6**), and α-pinene (**1.1.5**), which possess recognized antimicrobial and re-epithelization-inducing properties [36,49]. Furthermore, the sap was shown to block the lectin-dependent adhesion of bacterial pathogens, such as *Pseudomonas aeruginosa*, preventing biofilm-related complications that generally delay healing [36,69]. Traditional application in Hadhramout supported its wound healing properties, as ~90% of local users reported pronounced improvement of burn wounds treated with the plant [30].

### 6.6. Cardio-Protective and Vasorelaxant Activities

Diabetes raises the oxidative state, which results in several consequences, including cardiovascular effects. A high level of serum triglyceride and LDL cholesterol or low level of HDL cholesterol is linked with fatty depositions within the walls of arteries, with increased risk of heart attack and stroke.

Evaluations of the cardio-protective and vasorelaxant activities of *C. gileadensis* (Table 4) revealed the presence of multiple mechanisms, involving the activation of muscarinic receptors, modulation of vascular signaling pathways, and the induction of cardiovascular integrity markers [38,71,87]. In a study, the aerial part (branches) extract produced immediate, dose-related hypotensive and bradycardiac effects in rats through the activation of muscarinic cholinergic receptors, a response that was effectively inhibited by atropine sulfate [71]. At a molecular level, using a bio-guided assay, the diterpene resin acids, dehydroabietic acid (**1.3.1**) and sandaracopimaric acid (**1.3.5**), induced concentration-dependent relaxation of the phenylephrine (PE)-contracted pulmonary artery [38]. In particular, **1.3.1** was shown to enhance nitric oxide (NO) production via the PI3K/Akt-eNOS signaling pathway [38]. In diabetic models, treatment with the sap, methanol, or acetone extracts of the leaves and branches significantly improved cardiovascular health by inducing positive integrity markers such as adropin and NO and reducing malfunction markers including endothelin-1 (ET-1), vascular endothelial growth factor (VEGF), and cardiac enzymes like creatine kinase-MB (CK-MB) and lactate dehydrogenase (LD) [86]. These cardioprotective effects are further supported by a reduction in pro-inflammatory T-cells and an increase in Treg cells, which help prevent atheroma formation [86]. Additionally, the reported antioxidant properties of the plant further support these properties through mitigating oxidative stress-induced cardiovascular damage. For instance, the aerial parts extract inhibited xanthine oxidase (XO) and showed potent antioxidant activity, suggesting potential in preventing oxidative ischemic injury [11,86].

### 6.7. Antihyperlipidemic Activity

Hyperlipidemia is a pathological condition characterized by elevated levels of serum lipids due to genetic or lifestyle factors, representing a major risk factor for cardiovascular diseases. Diabetes further exaggerates this risk by disrupting lipid metabolism, resulting in increased total cholesterol (TC), triglycerides (TG), and low-density lipoprotein (LDL), and decreased high-density lipoprotein (HDL, good cholesterol) levels [100]. Recent evidence indicates that oxidative stress may contribute to the pathophysiology of hyperlipidemia [101].

Studies on the antihyperlipidemic potential of *C. gileadensis* showed its significant capacity to normalize lipid profiles in metabolic disorder models through reducing TC, TG, and LDL and augmenting HDL levels [45,79,86]. In hypercholesterolemic diabetic rats, aqueous extracts of the twigs and leaves effectively lowered TC, TG, and LDL, with the twigs extract demonstrating a notably higher therapeutic efficiency in restoring these parameters to near-normal levels [79]. This efficacy was further supported by the butanol fraction of the stem bark, which was shown to reduce cholesterol levels by enhancing the catabolism of LDL-C and its subsequent elimination as bile acids [45]. Guggulsterone (**2.1**), a key bioactive sterol in the leaves, was shown to act as an antagonist of the farnesoid X receptor (FXR), the primary bile acid receptor, thereby modulating systemic cholesterol metabolism and downregulating the bile salt export pump [44,102]. Furthermore, the sap and the aerial parts extract demonstrated significant hypolipidemic effects in Type 1 diabetic mice, with the sap demonstrating superior efficacy [86].

### 6.8. Gastroprotective and Anti-Ulcer Activities

*C. gileadensis* was shown to have gastroprotective and anti-ulcer properties through a dual mechanism, involving inhibition of gastric secretions and reinforcing the mucosal defensive barrier [74]. The aerial parts extract, administered at doses of 250 and 500 mg/kg, showed significant dose-dependent protection against gastric lesions induced by necrotizing agents (80% ethanol, 0.2 M NaOH, and 25% NaCl), hypothermic restraint stress, and indomethacin [74]. Histopathological evaluations demonstrated that pretreatment with the extract preserved the cytoarchitecture of gastric mucosa, preventing hemorrhagic necrosis and inflammatory cell infiltration. The extract was found to replenish depleted stomach wall mucus and maintain nonprotein sulfhydryl (NP-SH) concentrations, which potentially enhanced prostaglandin synthesis [74]. These effects are primarily attributed to bioactive constituents, such as flavonoids, saponins, and terpenoids (Table 1), through the prevention of free radicals and lipid peroxidation resulting from gastric mucosal damage [74,103,104]. Traditional usage surveys in Hadhramaut supported these gastroprotective properties, with local populations reporting the effective use of the plant for treating stomach ulcers and improving digestion [30,74].

### 6.9. Hepatoprotective Activity

Oxidative stress from toxins, infections, or diet disrupts hepatic homeostasis by damaging DNA, lipids, and proteins, initiating inflammatory pathways that exacerbate liver injury [105]. Hepatoprotective agents function by minimizing these harmful effects or restoring physiological mechanisms disturbed by hepatotoxins [76].

Promising results were reported by several studies, showing the ability of *C. gileadensis* to mitigate chemical-induced injury, strengthen antioxidant defense, and restore hepatic architecture [72,73,79]. The aerial parts and bark extracts showed significant protection against CCl_4_ and diethylnitrosamine (DEN)-induced-liver damage, as demonstrated by a marked reduction in serum AST, ALT, ALP, and bilirubin levels [72,73]. The reported hepatoprotective mechanisms involved free radical scavenging, reduction in MDA levels, and restoring endogenous antioxidants, including nonprotein sulfhydryls (NP-SH), GSH, and SOD [45,73,79]. Histopathological evaluations showed that the plant prevented confluent necrosis, steatosis, and inflammatory cell infiltration, maintaining the structural integrity of hepatocytes [72,73,79]. These therapeutic benefits are attributed to the rich phytochemical content of *C. gileadensis*, flavonoids, and volatile terpenes, which exhibit antioxidant activities promoting hepatocellular recovery [11,31,40].

### 6.10. Fertility-Enhancing Activity

In a study by Alhazmi (2025), *C. gileadensis* was shown to ameliorate infertility and erectile dysfunction in STZ-induced diabetic male mice [75]. The oral administration of the sap, methanol, and acetone extracts significantly reversed reproductive decline by significantly restoring testicular weight and increasing testosterone levels in diabetic and control male mice up to 70-fold and 30-fold, respectively, leaving FSH and LH unchanged, indicating a hypothalamic pituitary independent effect. The extracts also elevated the sperm count in addition to motility and percentage of normal sperm morphology. The sap remarkably showed a pronounced increase in testosterone level and sperm count. The plant was shown to exert these effects through the induction of positive erectile markers, such as NO and adropin, and suppression of the vasoconstrictor (negative erectile marker), endothelin [75]. It also reduced CD8^+^ and CD4^+^, which may decrease testes inflammation and induce its recovery in diabetic mice. Furthermore, the extracts restored testicular architecture and enhanced nitric oxide synthase (NOS) immunoreactivity in the testes. These therapeutic outcomes are attributed to the high steroid content and the presence of antioxidant and anti-inflammatory terpenes, which mitigated oxidative damage by reducing lipid peroxidation (LPO) and replenishing GSH, GSH-Px, and SOD levels in testicular tissue [75]. The steroidal derivative, guggulsterone (**2.1**), was reported to have multiple interactions, including an antagonizing effect on FXR and modulating the activity of other steroid receptors, such as estrogen receptor alpha, progesterone receptor, and pregnane X receptor (PXR) [106,107]. These interactions with key receptors involved in metabolic, reproductive, and steroidal hormone modulations can potentially influence fertility processes.

### 6.11. Antigenotoxic Activity

Genotoxicity refers to the capacity of physical, chemical, or biological agents to damage DNA, causing permanent and transmissible genetic alterations such as gene mutations and chromosomal abnormalities, which may lead to cancer, heritable defects, neuromuscular and neurodegenerative diseases, immune deficiencies, cardiovascular diseases, metabolic syndrome, aging, and infertility. Anti-genotoxic agents are substances capable of protecting DNA from damage or lessening existing harm [108,109].

Reported data (Table 4) showed that *C. gileadensis* could protect against chemical-induced damage, such as CCL_4_-induced genotoxicity and metabolic stress [32,45]. The dichloromethane extract reduced the proportion of chromosomal abnormalities in bone marrow cells and DNA fragmentation in hepatocytes [32]. This protective effect extended to the reproductive system, where the extract significantly lowered the frequency of sperm shape anomalies and chromosomal irregularities in spermatocytes [32]. In STZ-induced diabetic rats using the Comet assay, the butanol fraction of stem bark extract (100 mg/kg) reduced tailed DNA, associated with diabetic complications, from 13.00% to 11.00% and restored healthy untailed DNA to 89.00. Reactive oxygen species (ROS) affect DNA metabolism, leading to increased oxidative stress and inflammatory cascade activation that cause organ abnormalities. Therefore, antioxidants would protect the DNA structure [45]. Polyphenolic compounds were reported to have protective effects against DNA damage by reducing ROS and modulating enzymes responsible for the bioactivation of genotoxic agents. Free hydroxyl groups on the B ring (catechol moiety) and C-3 position of the C ring are important structural features for their antigenotoxic activity [108,110].

### 6.12. Immunomodulatory Activity

Immune reaction plays a critical role in inflammatory and atherosclerotic heart diseases. The immunomodulatory activity of *C. gileadensis* was mainly attributed to its ability to modulate cellular and humoral immune responses, as demonstrated in models of metabolic stress [79,86,87]. The sap and extracts of aerial parts modulated T-cell populations in diabetic mice, reducing the total lymphocytes and CD3^+^, CD4^+^, and CD8^+^ T-cell subsets, while increasing the Treg CD4^+^ CD25^+^ and CD8^+^ CD25^+^ cell subsets, which help regulate inflammatory cascades [86,87]. Furthermore, the aqueous extracts of the leaves and twigs were shown to restore humoral balance in diabetic rats by normalizing elevated immunoglobulins IgA, IgE, IgG, and IgM levels [79].

Plant polyphenols are recognized for their immunoregulatory effects, achieved by modulating immune cell functions and suppressing proinflammatory cytokine production and immune-related gene expression [111].

### 6.13. Antiaging Activity

Aging is a progressive decline in the organism’s ability to withstand stress, resulting in a gradual loss of physiological functions. It is driven largely by the accumulation of molecular damage in DNA, proteins, and lipids. Because oxidative reactions rise as intracellular ROS-scavenging capacity declines, antioxidants are thought to slow aging and may contribute to lifespan extension [78]. The anti-aging activity of *C. gileadensis* (Table 4) was evidenced by its capacity to extend the replicative lifespan of cellular models and mitigate oxidative stress [78]. The leaf extract (30 µg/mL), obtained by both ultrasonic-assisted and hydrodistillation methods, was shown to significantly increase the average lifespan of the K6001 yeast strain from 7.55 to 9.15 generations [78]. These longevity-promoting effects were attributed to the high terpenoid and phenolic contents of the plant, which function as powerful antioxidants that stabilize free radicals, protecting cells from oxidative damage [78]. The terpenic EO constituents of *C. gileadensis*, including α-pinene (**1.1.5**) and β-caryophyllene (**1.2.11**), are known for their antioxidant and anti-inflammatory activities, which may explain their potential geroprotective effects [11,78,112]. In particular, β-caryophyllene (**1.2.11**) was shown to extend lifespan in *Caenorhabditis elegans* by over 22%, primarily through reducing oxidative stress and modulating key stress-response genes including SIR-2.1, SKN-1, and DAF-16 [112]. Furthermore, the presence of flavonoids such as quercetin (**3.1.5**), which was shown to ameliorate aging processes, provided a complementary mechanism for cellular protection [113].

### 6.14. Analgesic and Antipyretic Activities

The extract of *C. gileadensis* showed promising analgesic and antipyretic properties (Table 4). In rodent (rats and mice) models, the aerial parts of the plant demonstrated potent dose-dependent peripheral and central analgesic effects. It significantly reduced acetic acid-induced writhing and increased latency in tail-flick and hot-plate tests [76,82]. At a 500 mg/kg dose, it showed superior potency compared to diclofenac, with 100% inhibition of writhing and 93.57% reduction in late-phase formalin-induced paw licking [82]. It also showed an ability to decrease yeast-induced hyperthermia in a dose-and-time dependent manner, indicating antipyretic action [76]. Moreover, a combination of the aerial parts extract (125 mg/kg) with a sub-therapeutic dose of the NSAID, diclofenac (12.5 mg/kg), resulted in a synergistic potentiation of the analgesic effect [82].

These therapeutic properties were mainly attributed to the triterpenes and flavonoids content of *C. gileadensis* [114,115], in particular, the sesquiterpene β-caryophyllene (**1.2.11**), which was recognized for its analgesic and anti-inflammatory potential [34].

### 6.15. Diuretic, Kidney Protective, and Antihyperuricemic Activities

The plant was traditionally utilized to treat urinary retention and promote the expulsion of renal calculi by increasing urine flow, in addition to its nephroprotective and antihyperurecemic effects, supported by pharmacological investigations, indicating potential diuretic and kidney protective activities (Table 4) [11,71,74]. The aerial parts extract was shown to significantly increase urine volume in a dose-dependent manner (22% to 31% increase) without significantly altering the excretion of Na^+^, K^+,^ and Ca^2+^ ions. This effect was potentially attributed to the inhibition of antidiuretic hormone (ADH) or blocking its receptors [76]. Beyond this diuretic action, nephroprotective effects were observed in metabolic stress in vivo models. Treatment with sap and aerial parts extracts reduced elevated serum urea, creatinine, and uric acid levels in diabetic and hypercholesterolemic models, as well as their ability to ameliorate histopathological damage, such as glomerular atrophy and tubular vacuolation, restoring near-normal renal architecture [76,79]. Since blood urea and serum creatinine are considered early biomarkers of diabetic nephropathy [116], the observed reductions in these parameters in diabetic mice suggest that *C. gileadensis* may exert renal protective effects and could slow the progression of diabetic nephropathy.

High levels of uric acid are closely linked to oxidative stress and the generation of ROS, a condition in which xanthine oxidase (XO) acts to convert xanthine to uric acid. Thus, reduced oxidative stress can improve urate handling and decrease tissue damage [11]. Although the aerial parts extract demonstrated weak in vitro inhibition of XO (IC_50_ = 251.2 µg/mL) compared to allopurinol (IC_50_ = 0.41 µg/mL); however, the involvement of multiple mechanisms, including antioxidant (DPPH and BCB assays), XO and COX-1 inhibitory activity, and inhibition of protein denaturation, could synergistically contribute to its nephroprotective effects [11]. These multiple mechanisms may provide scientific evidence for its use in treating gouty arthritis [17].

The nephroprotective activities are mostly attributed to a variety of phenolic and flavonoid compounds (Table 1), which act as powerful antioxidants, such as caffeic acid phenethyl ester (**4.2**), hesperetin (**3.3.2**), hesperidin (**3.3.3**), chrysin (**3.2.2**), gallic acid (**4.6**), rutin (**3.1.6**), and caffeic acid (**4.1**) [11]. They are also potent inhibitors of enzymes, such as cyclooxygenase (COX) and lipoxygenase (LOX), that control inflammation, a symptom characterizing gout and gouty arthritis [11].

### 6.16. Anticoagulant Activity

The sap, as well as the methanol and acetone extracts of branches and leaves of *C. gileadensis*, exhibited anticoagulative potential by significantly prolonging prothrombin time (PT), activated partial thromboplastin time (aPTT), and INR, which was more effective than heparin or aspirin. The observed effect was attributed to high levels of glycosaminoglycans [88].

Oxidative stress is considered an important factor causing thrombosis, due to the overproduction of reactive oxygen species (ROS) that can significantly impair the function of vascular endothelial cells, platelets, and red blood cells, leading to a cascade of events accelerating the formation of thrombi. Polyphenols and terpenes were reported to interfere with ROS-mediated platelet activation in thrombosis [117,118,119]. Accordingly, the anticoagulant activity of *C. gileadensis* may also be explained by the polyphenols and terpenes contents, which represent its main composition.

### 6.17. Antibacterial and Antibiofilm Activities

Numerous studies documented the in vitro and in vivo antibacterial and antibiofilm activities of different extract types, as well as EO of *C. gileadensis*, against a wide range of Gram-positive (Gram +ve) and Gram-negative (Gram −ve) bacteria, including multidrug-resistant pathogens, such as methicillin-resistant *Staphylococcus aureus* (MRSA), *P. aeruginosa*, and *Klebsiella pneumoniae* (Table 4) [23,33,39,49,70,80,81,85,89,90].

The bark and leaves extracts exhibited significant antibacterial activity against a wide spectrum of pathogens, including *S. aureus*, *P. aeruginosa*, and *K. pneumoniae* [23,85]. Notably, the antibacterial activity against *S. aureus* was shown to be superior to standard antibiotics, such as ampicillin [85]. Interestingly, bioactivity-guided phytochemical investigations of fresh stems identified a group of *ent*-verticillane-type diterpenoids, in particular, (9*S*,10*S*)-*ent*-9,10-epoxyverticillol (**1.3.3**), as the main antimicrobial agents against *K. pneumoniae*, potentially due to their ability to penetrate bacterial cell walls [39]. Furthermore, the EO demonstrated high antibacterial activity against *Bacillus subtilis* and *S. aureus*, which was attributed to potential bioactive compounds, such as β-pinene (**1.1.6**), β-caryophyllene (**1.2.11**), and terpinen-4-ol (**1.1.10**) [23,33,120]. Additionally, extracts derived from cell suspension cultures showed selective antibacterial action against *Staphylococcus epidermidis* [23]. Maqlam and Bin Kardous [70] developed two topical formulations: a cream and a gel, containing 10% of the bark extract. The gel formulation exhibited greater antibacterial activity against *S. aureus*, a common skin pathogen (15.7 mm inhibition zone diameter, IZD), compared to both the plant extract cream (IZD = 13.3 mm) and the standard cetrimide cream (IZD = 13.3 mm). This increased efficacy was attributed to the lower viscosity of the gel vehicle, which facilitated the release and diffusion of phenolic compounds into the surrounding medium compared to the cream base [70]. Moreover, a synergistic effect was reported between *C. gileadensis* extracts and some antibiotics, especially amoxicillin, polymixin B, and tetracycline [81].

Microorganisms use biofilm formation as a survival strategy to stick to surfaces and develop into organized communities covered in an extracellular polymeric matrix that they produce on their own. Compared to planktonic cells, this matrix confers significantly greater resistance to antibiotics and host immune defenses, protecting bacteria and encouraging chronic and persistent infections [121]. *C. gileadensis* demonstrated strong antibiofilm activities through interfering with bacterial cell adhesion and significantly reducing exopolysaccharide (EPS) content, which is a critical factor in biofilm stability [81]. The shoot extracts were shown to decrease EPS levels in *K. pneumoniae* by 39% and inhibit biofilm formation in multidrug-resistant strains of *Acinetobacter baumannii* [81]. Furthermore, the aqueous extract of branches (1 mg/mL) significantly killed more oral anaerobic biofilm-forming multispecies bacteria (unspecified), obtained from biofilms of subgingival and supragingival plaque from human donors, than 2% chlorhexidine [90]. Although the experiment utilized unknown bacterial isolates from plaque, the literature review of the thesis mentioned various bacterial strains typically associated with such infections, including *Enterococcus faecalis*, *Porphyromonas gingivalis*, *Fusobacterium nucleatum*, and *Streptococcus mutans* [90]. The major essential oil component, terpinene-4-ol (**1.1.10**), was identified as a potent antibiofilm agent against *S. aureus*, which was capable of preventing biofilm formation at sub-inhibitory concentrations and disrupting established biofilms, potentially through targeting penicillin-binding protein 2a [120].

### 6.18. Antiviral Activity

The leaf extract of *C. gileadensis* demonstrated potent and selective antiviral and virucidal activity against enveloped viruses, including herpes simplex virus type 2 (HSV-2) and respiratory syncytial virus type B (RSV-B). However, it was inactive against non-enveloped viruses, such as coxsackievirus B type 3 (CVB-3) and adenovirus type 5 (ADV-5) (Table 4) [44]. The suggested mechanism involved direct interaction between bioactive compounds in the extracts with receptor proteins on the viral envelope, inhibiting the virus’s ability to bind to host cells, eliminating its ability to cause infection. This activity was primarily attributed to the sterol guggulsterone (**2.1**), which was isolated from the leaf extract by TLC using a bio-guided method, and identified through HPLC-diode array (PDA) combined with electrospray ionization mass spectrometry (ESI-MS) [44]. Although the leaf extract exhibited selective antiviral properties, research evaluating the bark extract of *C. gileadensis* showed that it was ineffective as antiviral against a group of viruses, including hepatitis A virus (HAV-0), coxsackievirus, HSV-1, and HSV-2 [91].

Molecular docking and dynamics simulation studies were performed on guggulsterone against the dengue virus, targeting four proteins, including NS5 RNA-dependent RNA polymerase, dengue methyltransferase, NS3 protease-helicase, and dengue virus type 2 envelope glycoprotein. The virtual findings suggested that guggulsterone may be considered as a potent inhibitor of the dengue virus proteins and may be a significant inhibitor of the major envelope protein E of the dengue virus [102]. Notably, (-)-galbelgin (**5.3**), a lignan isolated from *Piper kadsura* (Choisy) Ohwi and detected in leaves of *C. gileadensis* was also reported as anti-human hepatitis B virus [122].

### 6.19. Antifungal Activity

Candidiasis has become the most common opportunistic invasive mycosis in the world due to its acute progression, difficulty in diagnosis, and high mortality rates, especially in immunocompromised individuals [85]. Although *Candida albicans* is commonly found in secondary endodontic infections at rates of up to 18%, the emergence of non-albicans species that show intrinsic resistance to traditional antifungal agents is increasingly impeding the success of treatment [85,90]. *C. gileadensis* has historically been used as an antiseptic to treat infected wounds. Several studies documented that extracts from different parts of this plant demonstrated broad-spectrum antifungal activity against various pathogenic yeasts and molds, including *C. albicans*, *C. glabrata*, *C. krusei*, *Cryptococcus neoformans*, and *Aspergillus niger* (Table 4) [33,37,85,89]. The leaf extracts effectively inhibited the growth of *C. albicans*, while the bark extracts remarkably demonstrated effectiveness against multidrug-resistant *Candida* species that were less sensitive to standard antifungals, such as itraconazole and voriconazole [23,85].

Phytochemical profiling of leaf extracts tentatively identified sordarin (**1.3.6**), an antifungal tetracyclic diterpenoid bearing a norbornene system, that was reported to inhibit fungal protein synthesis by targeting eukaryotic translation elongation factor 2 [40,123,124]. High levels of condensed tannins (proanthocyanidins) in the stem peels were believed to play a protective role against fungal pathogens by serving as a chemical defense mechanism [80]. It is worth noting that no specific studies were found describing the detection of proanthocyanidins in *C. gileadensis*. However, the occurrence of proanthocyanidins was reported in the decoction of *C. leptophloeos* bark [125,126]. Additionally, the EO contributed to the antifungal properties of *C. gileadensis* by selectively inhibiting *C. glabrata* and *C. krusei* [33]. Abbas, et al. [42] reported that syringic acid (**4.12**) isolated from the aerial parts of *C. gileadensis* exhibited moderate anticandidal activity, along with moderate antimalarial and antimycobacterial activities.

**Figure 19:**
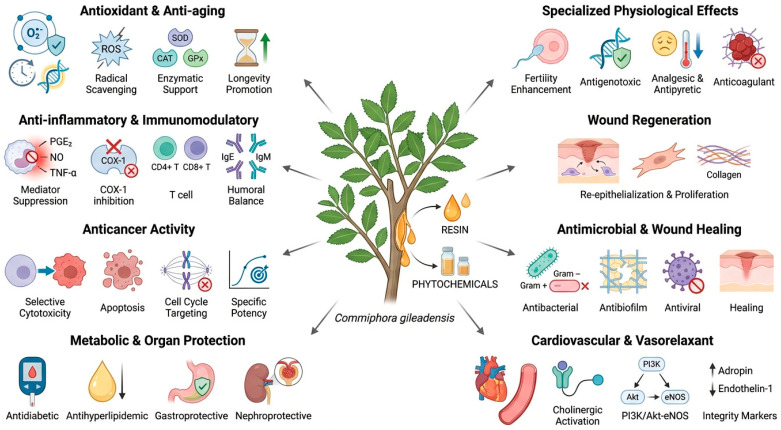
A representative diagram summarizing the pharmacological activities of *C. gileadensis*.

The pharmacological activities of *C. gileadensi* are summarized in Figure 19.

## 7. Pharmacokinetic and Toxicological Profiles of *C. gileadensis*

Although *C. gileadensis* was shown to have broad-spectrum biological activities and a rich phytochemical profile, making it a promising medicinal plant, little is known about its pharmacokinetic and toxicological properties. Even though a long history of traditional use suggests a favorable safety profile, there is a significant gap in the current literature, by focusing on the phytochemical composition and biological activities, with little presentation of thorough ADME and toxicological studies.

Reviewed publications either conducted sub-acute toxicological studies on various *C. gileadensis* extracts and administration doses and routes, with the oral route being the most popular, or preliminary acute toxicity tests with median lethal dose (LD_50_) determination in some cases. A summary of the reviewed toxicological studies is provided in Table 5.

*C. gileadensis* was generally categorized as practically non-toxic, and the reported acute oral lethal doses (LD_50_) were determined to be >2000 mg/kg in rats and >5000 mg/kg in mice [85,127]. Although single doses are well-tolerated, high-range doses (2500–5000 mg/kg) were associated with reversible sedation and a reduction in motor activity, possibly due to CNS-depressant effects of the high volatile oil content [85,127]. In a study evaluating the sub-acute toxicological effects (14 days at 1000 mg/kg/day), the extract did not adversely affect renal or hepatic function markers; however, significant dose-dependent reductions in hematological parameters (RBC, HCT, HGB, and WBC) suggested potential bone marrow inhibition on prolonged administration [127].

The ADME and toxicity profiles of the major bioactive sterol, guggulsterone (**2.1**), were predicted through in silico assessments [102]. Generally, the compound was concluded to be non-toxic, exhibiting no predicted hepatotoxic, immunotoxic, carcinogenic, cytotoxic, or mutagenic effects. It demonstrated high gastrointestinal absorption and blood–brain barrier (BBB) permeability. It was not a substrate for P-glycoprotein and exhibited a bioavailability of >40% in rats with a half-life of approximately 10 h [44,102]. It was predicted to inhibit CYP2C19 and CYP2C9 enzymes, with no inhibitory activity toward CYP1A2, CYP2D6, or CYP3A4 [102].

Another bioactive constituent, β-caryophyllene (**1.2.11**), was shown to selectively induce apoptosis in tumor cells without affecting normal human fibroblasts, indicating a good therapeutic window for medicinal applications [13,34].

## 8. Conclusions

*C. gileadensis*, historically known as the “Balm of Gilead,” possesses a remarkably diverse metabolic profile comprising over 170 secondary compounds, including terpenoids, flavonoids, and phenolic acids, among others, validating its traditional use, particularly as an effective antioxidant, anti-inflammatory, antimicrobial, and anticancer agent. However, because most current phytochemical evidence relies on tentative identifications from hyphenated techniques, such as GC-MS and LC-MS, future research must prioritize the phytochemical isolation and full structural elucidation of pure bioactive constituents to enable robust structure-activity relationship (SAR) analyses. Furthermore, scientific efforts should shift toward identifying specific molecular targets through detailed mechanistic studies and addressing critical gaps in the pharmacokinetic behavior (ADME) and long-term toxicological profile of the plant through thorough in vivo investigations. Therefore, the successful integration of *C. gileadensis* into modern evidence-based medicine requires a transition to human clinical trials for establishing safety and efficacy protocols. In addition, the expanded use of biotechnological methods, such as plant tissue culture, is essential to ensure a sustainable and standardized supply of bioactive metabolites, while safeguarding the species from ecological threats associated with overharvesting and climate change.

## Figures and Tables

**Table 1:** Secondary metabolites reported from different plant parts of *C. gileadensis*.

No. ^1^	Class/Name	CAS No. ^2^	Plant/Part							Ref.
			Aerial Parts	Stem	Leaf	Fruit	Bark	Resin/Balsam	Preparation Type ^3^	
1. **Terpenes and terpenoids**										
1.1. **Monoterpenes(oids)**										
**1.1.1**	*p*-Cymene	99-87-6	—	✓	✓	✓	—	✓	EO; resin	[12,20,30,31]
**1.1.2**	*p*-Cymen-8-ol	1197-01-9	✓	✓	✓	✓	—	—	Extract; EO	[11,12,32,33]
**1.1.3**	Limonene	138-86-3	—	✓	✓	✓	—	✓	EO; resin	[12,30]
**1.1.4**	*β*-Myrcene	123-35-3	—	✓	✓	✓	—	—	EO	[12,31]
**1.1.5**	*α*-Pinene	80-56-8	—	✓	✓	✓	—	—	EO	[12,20,31,34]
**1.1.6**	*β*-Pinene	127-91-3	—	✓	✓	✓	—	—	EO	[12,20,31]
**1.1.7**	*β*-Phellandrene	555-10-2	—	✓	✓	✓	—	—	EO	[12,31]
**1.1.8**	Sabinene	3387-41-5	—	✓	✓	✓	—	✓	EO; resin	[12,20,30,31,34]
**1.1.9**	*γ*-Terpinene	99-85-4	—	✓	✓	✓	—	—	EO	[12,31,34]
**1.1.10**	Terpinen-4-ol	562-74-3	✓	✓	✓	✓	—	—	EO	[12,20,31,33,34]
**1.1.11**	α-Thujene	2867-05-2	✓	—	✓	✓	—	✓	EO; resin	[20]
**1.1.12**	*β*-Thujene	28634-89-1	✓	✓	✓	✓	—	—	EO; Extract	[32,34]
1.2. **Sesquiterpenes(oids)**										
**1.2.1**	5-Acetoxy-2-methoxyfuranogermacr-1(10)-en-6-one	1809980-25-3	—	—	—	—	—	✓	Extract	[35]
**1.2.2**	Alloaromadendrene oxide	85710-39-0	✓	—	—	—	—	—	Extract	[32]
**1.2.3**	Cadalene	483-78-3	✓	—	—	—	—	—	Extract; EO	[32,33]
**1.2.4**	β-Cadinene	523-47-7	✓	—	—	—	—	—	Extract	[11]
**1.2.5**	δ-Cadinene	483-76-1	✓	—	—	—	—	—	EO	[33]
**1.2.6**	*τ*-Cadinol	5937-11-1	✓	—	—	—	—	—	Extract	[11,32]
**1.2.7**	*α*-Cadinol	481-34-5	✓	—	—	—	—	—	EO	[33]
**1.2.8**	*α*-Calacorene	21391-99-1	✓	—	—	—	—	—	EO	[33]
**1.2.9**	*cis*-Calamenene	72937-55-4	✓	✓	—	—	—	—	EO	[33,36]
**1.2.10**	*Trans*-Calamenene	40772-39-2	✓	—	—	—	—	—	Extract	[11]
**1.2.11**	*β*-Caryophyllene	87-44-5	✓	✓	✓	✓	—	—	Extract; EO	[11,20,34,36]
**1.2.12**	Cedrol	77-53-2	✓	—	—	—	—	—	Extract	[11]
**1.2.13**	*β*-Copaene	18252-44-3	✓	—	✓	✓	—	—	Extract; EO	[11,34]
**1.2.14**	α-Cubebene	17699-14-8	—	✓	—	—	—	—	Extract	[37]
**1.2.15**	Cubenene	38758-02-0	✓	—	—	—	—	—	Extract	[11]
**1.2.16**	Cubenol	21284-22-0	✓	—	—	—	—	—	Extract	[32,33]
**1.2.17**	1-*epi*-Cubenol	21284-22-0	✓	—	—	—	—	—	Extract	[11]
**1.2.18**	4*α*,10*α*-Dihydroxy-1α,5α*H*-guaia-6-ene	74513-46-5	—	—	—	—	—	✓	Extract	[35]
**1.2.19**	7*α*,11-Dihydroxycadin-10(14)-ene	658062-23-8	—	—	—	—	—	✓	Extract	[35]
**1.2.20**	*β*-Elemene	515-13-9	✓	—	—	—	—	—	Extract	[11]
**1.2.21**	*β*-Eudesmol	473-15-4	✓	—	—	—	—	—	Extract; EO	[11,32,33]
**1.2.22**	Epiglobulol		✓	—	—	—	—	—	Extract	[11]
**1.2.23**	Germacrene D	23986-74-5	✓	✓	✓	✓	—	—	Extract; EO	[12,20,33,34]
**1.2.24**	Globulol	489-41-8	✓	—	—	—	—	—	Extract	[11]
**1.2.25**	Humulene	6753-98-6	—	✓	—	—	—	—	EO	[36]
**1.2.26**	Humulene epoxide-II	19888-34-7	✓	—	—	—	—	—	EO	[33]
**1.2.27**	*γ*-Himachalene	66322-03-0	✓	—	—	—	—	—	Extract	[32]
**1.2.28**	(1(10)*E*,2*R*,4*R*)-2-Methoxy-8,12-epoxygermacra-1(10),7,11-tetraen-6-one	2677757-79-6	—	—	—	—	—	✓	Extract	[35]
**1.2.29**	(1*E*)-3-Methoxy-8,12-epoxygermacra-1,7,10,11-tetraen-6-one	NA	—	—	—	—	—	✓	Extract	[35]
**1.2.30**	*α*-Muurolene	17627-24-6	✓	—	—	—	—	—	Extract	[11]
**1.2.31**	*γ*-Muurolene	30021-74-0	✓	—	—	—	—	—	Extract	[11]
**1.2.32**	T-Muurolol	19912-62-0	✓	—	—	—	—	—	EO	[33]
**1.2.33**	Myrrhanolide D	NA	—	—	—	—	—	✓	Extract	[35]
**1.2.34**	Myrrhasin A	NA	—	—	—	—	—	✓	Extract	[35]
**1.2.35**	Myrrhone	183551-83-9	—	—	—	—	—	✓	Extract	[35]
**1.2.36**	Selina-6-en-4-ol	2247465-38-7	✓	—	—	—	—	—	Extract	[32]
**1.2.37**	Sesquisabinene hydrate	NA	✓	—	—	—	—	—	Extract	[32]
**1.2.38**	Spathulenol	6750-60-3	✓	—	—	—	—	—	Extract; EO	[32,33]
**1.2.39**	*β*-Selinene	17066-67-0	✓	—	—	—	—	—	Extract	[11]
**1.2.40**	Veridiflorol	552-02-3	✓	—	—	—	—	—	Extract	[32,33]
1.3. **Diterpenes(oids)**										
**1.3.1**	Dehydroabietic acid	1740-19-8	—	—	—	—	—	✓	Extract	[38]
**1.3.2**	(13*S*,14*S*)-*Ent*-13,14-Epoxyverticillol	NA	—	✓	—	—	—	—	Extract	[31,39]
**1.3.3**	(9*S*,10*S*)-*Ent*-9,10-Epoxyverticillol	NA	—	✓	—	—	—	—	Extract	[31,39]
**1.3.4**	Gileadenol	NA	—	✓	—	—	—	—	Extract	[31,39]
**1.3.5**	Sandaracopimaric acid	471-03-4	—	—	—	—	—	✓	Extract	[38]
**1.3.6**	Sordarin	107870-29-7	—	—	✓	—	—	—	Extract	[40]
**1.3.7**	(1*S*,3*E*,7*E*,11*R*)-(+)-Verticilla-3,7,12(18)-triene	870706-62-0	—	✓	—	—	—	—	Extract; EO	[31,36,39]
**1.3.8**	*Ent*-Verticillol	129707-30-8	—	✓	—	—	—	—	Extract	[31,39]
**1.3.9**	Verticiol	NA	—	—	—	—	✓	—	Extract	[11,12,31]
1.4. **Triterpenes(oids)**										
**1.4.1**	1*α*-Acetoxycycloartan-24-ene-2α,3*β*-diol	1005517-03-2	—	—	—	—	—	✓	Extract	[41]
**1.4.2**	3*β*-Acetoxycycloartan-24-ene-1*α*,2*α*-diol	1005517-02-1	—	—	—	—	—	✓	Extract	[41]
**1.4.3**	Canophyllal	14440-40-5	✓	✓	—	—	—	—	Extract	[39,42]
**1.4.4**	Commigileadin A	NA	✓	—	—	—	—	—	Extract	[43]
**1.4.5**	Cycloartan-23*E*-ene-1α,2α,3β,25-tetrol	NA	—	—	—	—	—	✓	Extract	[41]
**1.4.6**	Cycloartan-24-ene-1*α*,2*α*,3*α*-triol	1005517-01-0	—	—	—	—	—	✓	Extract	[41]
**1.4.7**	Cycloartan-24-ene-1*α*,2*α*,3*β*-triol	942407-97-8	—	—	—	—	—	✓	Extract	[41]
**1.4.8**	Cycloartan-24-ene-1*α*,3*β*-diol	1005517-05-4	—	—	—	—	—	✓	Extract	[41]
**1.4.9**	24*R*,25-Epoxycycloartane-1*α*,2*α*,3*β*-triol	NA	—	—	—	—	—	✓	Extract	[41]
**1.4.10**	24*S*,25-Epoxycycloartane-1*α*,2*α*,3*β*-triol	NA	—	—	—	—	—	✓	Extract	[41]
**1.4.11**	3*β*-Isovaleroyloxycycloartan-24-ene-1*α*,2*α*-diol	1005517-04-3	—	—	—	—	—	✓	Extract	[41]
**1.4.12**	Friedelin	559-74-0	✓	✓	—	—	—	—	Extract	[39,42]
**1.4.13**	Oleanonic acid	17990-42-0	✓	—	—	—	—	—	Extract	[42]
**1.4.14**	Oleanonic aldehyde	33608-08-1	—	✓	—	—	—	—	Extract	[39]
**1.4.15**	Urs-12-en-3-one-28-al	35936-63-1	—	✓	—	—	—	—	Extract	[39]
2. **Phytosterols**										
**2.1**	Guggulsterone	95975-55-6	—	—	✓	—	—	—	Extract	[44]
**2.2**	Stigmasterol	83-48-7	✓	—	—	—	—	✓	Extract	[35,43]
3. **Flavonoids**										
3.1. **Flavonols and their derivatives**										
**3.1.1**	Kaempferol	520-18-3	✓	—	—	—	✓	—	Extract	[43,45,46]
**3.1.2**	Mearnsetin	16805-10-0	✓	—	—	—	✓	—	Extract	[42,46]
**3.1.3**	Myricetin	529-44-2	—	—	—	—	✓	—	Extract	[45]
**3.1.4**	Pinoquercetin	491-49-6	—	—	—	—	—	—	Tissue culture	[23]
**3.1.5**	Quercetin	117-39-5	✓	—	✓	—	✓	—	Extract	[25,32,40,42,43,45]
**3.1.6**	Rutin	153-18-4	✓	—	—	—	—	—	Extract	[11]
**3.1.7**	3,4′,5,7-Tetrahydroxy-3′,6′-dimethoxyflavone	127615-73-0	—	—	—	—	✓	—	Extract	[45]
3.2. **Flavones** **and their derivatives**										
**3.2.1**	Apigenin	520-36-5	✓	—	—	—	✓	—	Extract	[11,45]
**3.2.2**	Chrysin	480-40-0	✓	—	—	—	—	—	Extract	[11]
**3.2.3**	Isoorientin	4261-42-1	—	—	—	—	—	—	Tissue culture	[23]
**3.2.4**	Isovitexin	38953-85-4	—	—	—	—	—	—	Tissue culture	[23]
**3.2.5**	Orientin	28608-75-5	—	—	—	—	—	—	Tissue culture	[23]
**3.2.6**	Saponarin	20310-89-8	—	—	✓	—	—	—	Extract	[40]
3.3. **Flavanones** **and their derivatives**										
**3.3.1**	Eriodictyol	552-58-9	—	—	—	—	—	—	Tissue culture	[23]
**3.3.2**	Hesperetin	520-33-2	✓	—	—	—	—	—	Extract	[11]
**3.3.3**	Hesperidin	520-26-3	✓	—	—	—	—	—	Extract	[11]
**3.3.4**	Homoeriodictyol	446-71-9	—	—	—	—	—	—	Tissue culture	[23]
**3.3.5**	Isosakuranetin; Naringenin-4′-methyl ether	480-43-3	✓	—	—	—	✓	—	Extract	[43,47]
**3.3.6**	Naringenin	480-41-1	✓	—	—	—	✓	—	Extract	[11,43,45]
3.4. **Isoflavones** **and their derivatives**										
**3.4.1**	Daidzein	486-66-8	—	—	—	—	✓	—	Extract	[45]
**3.4.2**	Glycitein	40957-83-3	—	—	—	—	✓	—	Extract	[45]
3.5. **Flavan** **-3-ols and their derivatives**										
**3.5.1**	Catechin	154-23-4	✓	—	—	—	—	—	Extract	[11]
**3.5.2**	Catechin Gallate	25615-05-8	—	—	—	—	—	—	Tissue culture	[23]
3.6. **Dihydrochalcone derivatives**										
**3.6.1**	Phloretin	60-82-2	—	—	—	—	✓	—	Extract	[45]
3.7. **Prenylated** **flavonoids**										
**3.7.1**	Comophorin A	2116562-68-4	—	—	—	—	✓	—	Extract	[46,47]
**3.7.2**	Comophorin B	2116562-69-5	—	—	—	—	✓	—	Extract	[45,46,47]
**3.7.3**	Comophorin C	2116562-70-8	—	—	—	—	✓	—	Extract	[45,46,47]
**3.7.4**	Comophorin D	2116562-71-9	—	—	—	—	✓	—	Extract	[46,47]
**3.7.5**	Comophorin E	2116562-73-1	—	—	—	—	✓	—	Extract	[45,46,47]
**3.7.6**	Comophoroside A	2116562-72-0	—	—	—	—	✓	—	Extract	[46,47]
**3.7.7**	2-(3,5-Dihydroxy-4-methoxyphenyl)-3,5-dihydroxy-8,8-dimethyl-7,8-dihydropyrano [3,2 g] chromen-4 (6*H*)-one	2115706-97-1	—	—	—	—	✓	—	Extract	[46,47]
4. **Phenolic acids and their derivatives**										
**4.1**	Caffeic acid	331-39-5	—	—	—	—	✓	—	Extract	[45]
**4.2**	Caffeic acid phenethyl ester	104594-70-9	✓	—	—	—	—	—	Extract	[11]
**4.3**	Cinnamic acid	621-82-9	—	—	—	—	✓	—	Extract	[45]
**4.4**	Ellagic acid	476-66-4	—	—	—	—	✓	—	Extract	[45]
**4.5**	Ferulic acid	1135-24-6	—	—	—	—	✓	—	Extract	[45]
**4.6**	Gallic acid	149-91-7	✓	—	—	—	✓	—	Extract	[11,45]
**4.7**	Hydro-p-coumaric acid; Phloretic acid	501-97-3	—	—	—	—	✓	—	Extract	[45]
**4.8**	4-Hydroxybenzoic acid	99-96-7	—	—	—	—	✓	—	Extract	[45]
**4.9**	3-*O*-Methyl gallic acid	3934-84-7	—	—	—	—	✓	—	Extract	[45]
**4.10**	Protocatechuic acid	99-50-3	—	—	—	—	✓	—	Extract	[45]
**4.11**	Sinapic acid	530-59-6	—	—	—	—	✓	—	Extract	[45]
**4.12**	Syringic acid	530-57-4	✓	—	—	—	—	—	Extract	[42]
5. **Lignans**										
**5.1**	Anthricin	19186-35-7	—	—	✓	—	—	—	Extract	[40]
**5.2**	*β*-Conidendrin	5474-93-1	—	—	✓	—	—	—	Extract	[40]
**5.3**	(-)-Galbelgin	10569-12-7	—	—	✓	—	—	—	Extract	[40]
**5.4**	Justicidin B	17951-19-8	—	—	—	—	—	—	Tissue culture	[23]
**5.5**	Podophyllotoxin	518-28-5	—	—	✓	—	—	—	Extract	[40]
6. **Coumarins**										
**6.1**	6,7-Dihydroxycoumarin; Aesculetin	305-01-1	—	—	—	—	✓	—	Extract	[45]
**6.2**	Umbelliferone	93-35-6	—	—	—	—	✓	—	Extract	[45]
7. **Fatty acid and lipids**										
**7.1**	8-Aminocaprylic acid	1002-57-9	—	—	✓	—	—	—	Extract	[40]
**7.2**	Arachidic acid	506-30-9					✓	✓	Extract; Commercial balsamic oil	[45,48]
**7.3**	Behenic acid	112-85-6	—	—	—	—		✓	Commercial balsamic oil	[48]
**7.4**	Capric acid	334-48-5	—	—	—	—		✓	Commercial balsamic oil	[48]
**7.5**	Caproic acid	142-62-1	—	—	—	—		✓	Commercial balsamic oil	[48]
**7.6**	Caprylic acid	124-07-2	—	—	—	—		✓	Commercial balsamic oil	[48]
**7.7**	Ceramide	NA	✓	—	—	—	—	—	Extract	[49]
**7.8**	*cis*-11-Eicosenoic acid	62322-84-3	—	—	—	—		✓	Commercial balsamic oil	[48]
**7.9**	Elaidic acid	112-79-8	—	—	—	—		✓	Commercial balsamic oil	[48]
**7.10**	α-Linolenic acid	463-40-1	—	—	—	—	—	✓	Commercial balsamic oil	[48]
**7.11**	γ-Linolenic acid	506-26-3	—	—	—	—		✓	Commercial balsamic oil	[48]
**7.12**	Heptadecanoic acid	506-12-7	—	—	—	—		✓	Commercial balsamic oil	[48]
**7.13**	Hexosylceramide	NA	✓	—	—	—	—	—	Extract	[49]
**7.14**	Lauric acid	143-07-7	—	—	—	—		✓	Commercial balsamic oil	[48]
**7.15**	Lignoceric acid	557-59-5	—	—	—	—		✓	Commercial balsamic oil	[48]
**7.16**	Linoleic acid	60-33-3	—	—	—	—		✓	Commercial balsamic oil	[48]
**7.17**	Myristic acid	544-63-8	—	—	—	—		✓	Commercial balsamic oil	[48]
**7.18**	Myristoleic acid	544-64-9	—	—	—	—		✓	Commercial balsamic oil	[48]
**7.19**	Oleic acid	112-80-1	—	—	—	—		✓	Commercial balsamic oil	[48]
**7.20**	Palmitic acid; Pentadecanoic acid	57-10-3	—	✓	—	—		✓	Extract; Commercial balsamic oil	[37,48]
**7.21**	Palmitoleic acid	373-49-9	—	—	—	—	✓	✓	Extract; Commercial balsamic oil	[45,48]
**7.22**	Phosphatidylethanolamine	1190-00-7	✓	—	—	—	—	—	Extract	[49]
**7.23**	Stearic acid	57-11-4	—	—	—	—	✓	✓	Extract; Commercial balsamic oil	[45,48]
8. **Alkaloids**										
**8.1**	10-Hydroxycamptothecin	19685-09-7	—	—	—	—	—	—	Tissue culture	[23]
**8.2**	Laudanosine	2682-85-7	—	—	—	—	—	—	Tissue culture	[23]
9. **Miscellaneous group**										
**9.1**	*p*-Coumaroylputrescine	34136-53-3	—	—	—	—	✓	—	Extract	[45]
**9.2**	2,6-Deoxyfructosazine	36806-15-2	—	—	✓	—	—	—	Extract	[40]
**9.3**	Di-(2-ethylhexyl)-phthalate	117-81-7	✓	—	—	—	—	—	Extract	[32]
**9.4**	Dimethylmalonic acid, 4-acetylphenyl ethyl ester	NA	—	—	✓	—	—	—	Extract	[40]
**9.5**	Eicosane	112-95-8	✓	—	—	—	—	—	Extract	[32]
**9.6**	Eugenol	97-53-0	✓	✓	—	—	—	—	EO	[33,36]
**9.7**	Folic acid	59-30-3	—	✓	—	—	—	—	Extract	[37]
**9.8**	Glutaric acid, tridec-2-yn-1-yl 3-nitrobenzyl ester	NA	—	—	✓	—	—	—	Extract	[40]
**9.9**	5-Methyl-2(5*H*)-furanone	591-11-7	—	✓	—	—	—	—	Extract	[37]
**9.10**	Nonadecane	629-92-5	✓	—	—	—	—	—	Extract	[32]
**9.11**	Nonane	111-84-2	✓	—	—	—	—	—	Extract	[11]
**9.12**	Oxydisuccinic acid	84852-72-2	—	—	—	—	✓	—	Extract	[45]
**9.13**	Sebacic acid, octyl 1-phenylpropyl ester	NA	—	—	✓	—	—	—	Extract	[40]
**9.14**	Quinic acid	77-95-2	—	—	—	—	✓	—	Extract	[45]
**9.15**	Succinic acid	110-15-6	—	—	—	—	✓	—	Extract	[45]
**9.16**	Vitamin B1	59-43-8	—	✓	—	—	—	—	Extract	[37]

^1^ Bold font indicates compound codes; ^2^ NA: CAS number is not available in the Reaxy database; ^3^ EO: Essential oil; “—” = not detected; “✓” = detected.

**Table 2:** Comparative summary of the major chemical components reported for *C. gileadensis* across different geographical origins, plant parts, and preparation methods.

Geographical Origin	Plant Part	Preparation Method	Main Components (Relative %)	Ref.
Makkah, Saudi Arabia	Dried aerial parts	Hydrodistillation (3 h)	α-Cadinol **1.2.7** (10.1%), spathulenol **1.2.38** (5.8%), viridiflorol **1.2.40** (4.9%)	[33]
Makkah, Saudi Arabia	Fresh aerial parts	Hydrodistillation (3 h)	α-Calacorene **1.2.8** (9.4%), terpinen-4-ol **1.1.10** (8.5%), δ-cadinene **1.2.5** (5.0%)	[33]
Makkah, Saudi Arabia	Fresh flowering tops	Hydrodistillation (3 h)	Terpinen-4-ol **1.1.10** (9.8%), cadalene (5.4%), δ-cadinene **1.2.5** (4.8%)	[33]
Medina (Badr), Saudi Arabia	Fresh aerial parts	Steam distillation (4 h)	β-Myrcene **1.1.4** (17.44%), nonane **9.11** (10.88%), verticiol **1.3.9** (10.56%), β-phellandrene **1.1.7** (9.59%)	[11]
Medina (Badr), Saudi Arabia	Fresh aerial parts	70% Ethanolic extract	Copaene **1.2.13** (11.48%), α-muurolene **1.2.30** (9.01%), β-selinene **1.2.39** (5.02%)	[11]
Almog, Middle East (Native KSA)	Fresh aerial parts	Steam distillation (3 h)	Sabinene **1.1.8** (22.7%), terpinen-4-ol **1.1.10** (18.7%), α-pinene **1.1.5** (14.4%), cymene **1.1.1** (13.6%)	[20]
Almog, Middle East (Native KSA)	Resin/exudate	Direct injection	Sabinene **1.1.8** (43.8%), α-pinene **1.1.5** (24.0%), β-pinene **1.1.6** (6.3%)	[20]
Almog, Middle East (Native KSA)	Resin/exudate	Static Headspace Solid Phase microextraction (HS-SPME)	Sabinene **1.1.8** (46.4%), α-pinene **1.1.5** (25.8%), α-thujene **1.1.11** (4.3%)	[20]
Ein Gedi, Middle East	Leaves and Fruits	Methyl-*tert*-butyl ether (MTBE) extraction	Sabinene **1.1.8** (21.11%), β-caryophyllene **1.2.11** (20.12%), germacrene D **1.2.23** (19.62%)	[34]
Khulais, Saudi Arabia	Shoots/aerial parts	Successive solvent extraction	β-Eudesmol **1.2.21** (11.9%), veridiflorol **1.2.40** (5.41%), spathulenol **1.2.38** (5.18%)	[32]
Breiman, Saudi Arabia	Shoots/aerial parts	Successive solvent extraction	γ-Himachalene **1.2.27** (21.43%), di-(2-ethylhexyl)-phthalate **9.3** (15.18%), veridiflorol **1.2.40** (9.63%)	[32]
Imported (India)	Resin/exudate	Reflux extraction with ethyl acetate	Isolated sesquiterpenoids, including myrrhanolide D **1.2.33**, myrrhasin A **1.2.34**	[35]

**Table 3:** Summary of traditional medicinal uses and ethnobotanical applications of various plant organs and preparations from *C. gileadensis* by different countries and populations.

Traditional Medicinal Use	Plant Material	Country/Population	Ref.
– As an antiseptic and wound healing – Treatment for headaches, cataracts, and blurred vision – For paralysis, stroke, hearing disorders, and mending fractures – For weight reduction, gastrointestinal ailments, and arthritis – As a contraceptive, for cervical infections – As an antidote for snake bites and scorpion stings	Sap or resin	Ancient Arabia, Palestine, Yemen, Saudi Arabia	[11,23,69,73,75]
– As an antiseptic for infected wounds and injuries – Juice from ground bark treated allergic skin reactions, burns, eczema, and inflammation – Aqueous extracts are utilized as an anti-hypertensive	Bark	Saudi Arabia, Oman (Dhofar tribes), Yemen	[33,37,70,71,73]
– Decoctions are used as analgesics (pain relief), laxatives (constipation), and diuretics (urine output) – To expel renal calculi – Crushed leaves are applied to treat eye tumors – General treatment for bacterial infections and inflammation	Leaves and flowers	Palestine, Arab populations, Yemen	[17,71,73,76,77]
– Preparations used for chest, stomach, and kidney complaints – Promoted digestion and treated jaundice, scurvy, and rheumatism – Wood is utilized for incense, perfumes, and medicinal products – Believed to improve memory	Seeds and wood	Saudi Arabia, Yemen, Egypt	[69,72,73,75]
– Employed as a natural toothbrush (miswak) for oral hygiene – Boiled in water to extract oil for treating traumatic injuries, fractures, and falls from high places	Twigs, branches, and stems	Saudi Arabia, Oman, Ancient Middle East	[13,33,37,69]
– Indicated for “cold” phlegm disorders (epilepsy, tetanus, and gonorrhea) – For ear problems, excess phlegm (catarrh), backaches, and knee pain – To massage arthritic joints	EO	Saudi Arabia, Yemen, Syria, Egypt	[40,44,69]
– General treatment for obesity, inflammatory diseases, coronary artery disease, and rabies – As a cleansing bath for newborns – For sore throat, laryngitis, cough, fever, and swelling	Total plant extracts	Saudi Arabia, Oman, Sudan, Ayurvedic Medicine	[23,32,37,45]

**Table 4:** Biological activities reported for *C. gileadensis*.

Biological Activity	Plant Part Used	Test Type/Model	Main Result/Outcome	Ref.
1. **Antioxidant**	- Leaves	- DPPH free radical scavenging assay and determination of total phenolic content (TPC)	- High antioxidant - Extraction by the ultrasonic-assisted extraction (USE) method showed antioxidant DPPH scavenging activity more than the hydrodistillation extraction method (HDE) extracts by about 3.3% (77.78% vs. 75.27%) and yielded higher TPC (118.71 vs. 101.47 mg GAE/g DM).	[78]
	- Aerial parts	- DPPH free radical scavenging assay and β-carotene bleaching (BCB)	- Steam-distilled EO showed stronger antioxidant activity than the ethanolic extract, with IC_50_ values of 56.5 ± 0.4 and 22.2 ± 0.5, respectively, compared to ascorbic acid (1.25 ± 0.05 µg/mL). - The extract showed remarkable BCB inhibition IC_50_ of 75.8 µg/mL.	[11]
	- Leaves and twigs	Alloxan-induced diabetic hypercholesterolemic rats and the activity of the following: - Enzymes estimated in the liver tissue homogenate, including SOD, CAT, and GST - Lipid peroxidation through estimating the concentration of malondialdehyde (MDA)	- Extracts enhanced the activities of CAT, SOD, and GST while significantly (*p* < 0.001) reducing MDA levels compared with the positive control. The aqueous twig extract exhibited greater efficacy than the leaf extract in restoring antioxidant enzyme activities toward normal levels.	[79]
	- Leaf and stem peel	- DPPH, ABTS, and H_2_O_2_ free radical scavenging assays	- Methanol ex-tract of stem peel showed more potent activity than leaves in all assays, including DPPH (EC_50_ 1.06 vs. 3.39 µg/mL); ABTS (EC_50_ 0.55 vs. 0.69 µg/mL); H_2_O_2_ (EC_50_ 1.28 vs. 2.43 µg/mL).	[80]
	- Stem bark	- STZ-induced diabetic rats (GSH, SOD, MDA)	- *n*-Butanol fraction (100 mg/kg) significantly improved endogenous defenses as GSH increased by 82.51%, SOD by 49.03%, and lipid peroxidation (MDA) was reduced by 33.41% compared to diabetic controls.	[45]
	- Stems	- DPPH and FRAP (Ferric Reducing Antioxidant Power) assays	- Methanolic extract showed moderate DPPH scavenging activity (>50% at 1 mg/mL) and high FRAP reducing power (1.95 mM FE/mg), comparable to ascorbic acid (2.1 mM FE/mg).	[81]
2. **Anti-inflammatory**	- Aerial parts	- Carrageenan-induced paw edema in rats (acute inflammation). - Cotton-pellet granuloma method (chronic inflammation).	- Ethanol extract (250 and 500 mg kg^−1^ body weight orally) significant reduction in paw edema. - Significantly reduced the granuloma formation in rats	[76]
	- Aerial parts	- COX-1 inhibitory activity and protein denaturation	- At a concentration of 450 μg/mL, the ethanol extract displayed COX-1 inhibitory activity. - Inhibited protein denaturation (IC_50_ 110.5 µg/mL).	[11]
	- Aerial parts	- Paw edema induced by carrageenan (acute inflammation) - Granuloma induced by cotton pellet in mice (chronic inflammation) - Testing the anti-inflammatory effect of a combination of reduced doses of extract and diclofenac.	- Paw edema induced by carrageenan was significantly suppressed (73.9% inhibition) by methanol extract in comparison to diclofenac at the first hour. - Significant weight reduction in granuloma tissue (85.99% reduction). - The combination resulted in synergistic potentiation of anti-inflammatory effects. - Potency was equivalent to diclofenac.	[82]
	- Aerial parts	- Carrageenan-induced paw edema in mice	- Methanol extract (500 mg/kg) significantly reduced PGE_2_ (40.8%), NO (55.47%), and TNF-α (19%) at the site of inflammation.	[83]
	- Leaves	- NF-κB pathway inhibition in vitro	- Isolated guggulsterone acts as an antagonist of the bile acid receptor and exerts potent anti-inflammatory effects via NF-κB suppression.	[44]
3. **Anticancer**	- Polysaccharides from freshly harvested stems.	- Apoptosis assay colorectal carcinoma cell lines SW480, SW620	- Potent antiproliferative activity, - Several doses over 24 h showed: - Against SW480: significant increase in both early and late apoptotic areas (Annexin V+/PI+) with IC_50_ 13.15 µg/mL - Against SW620: notable increase in the late apoptotic region IC_50_ 32.02 µg/mL.	[84]
	- Leaves, seeds, callus, cell suspension	- Cytotoxic assay against: - Human breast adenocarcinoma (MCF-7). - Human epithelial pancreas carcinoma (PANC-1). - Human prostate adenocarcinoma, grade IV (PC-3). Human epithelial lung carcinoma (A549). - Fibroblasts. - Normal skin cells (CCD-1064SK). - Compared to normal human fibroblast cell lines.	- Selective activity of the methanol extracts against a specific cell line) - Leaf extract showed: - Broad cytotoxic effect against all tested cell lines. - The strongest cytotoxicity against A549. - But it has clear toxic effects on fibroblast cells. - Only A549 was sensitive to all extracts. - The seed extract was more effective than the callus extract against the PanC1 cell line. - No effect of cell suspension extract	[23]
	- Sap	- Antiproliferative activity on: - Immortalized keratinocytes. - Human dermal fibroblasts. - Human dermoid carcinoma cells - Human ex vivo skin cultures.	- Ethanol extract significantly reduced cell viability of both immortalized and transformed epidermal cells by 64% and 68%, respectively. - Normal fibroblasts and human skin organ cultures were protected from this effect. - The induced apoptosis did not take place in the cells in the pre-replicative (G_1_ phase), only at the later phases involved in DNA replication and cell division.	[13]
	- Aerial parts	- in vitro cytotoxicity against the HepG2 cell line	- HDE showed significant anticancer activity against HepG2 cells; a dose of 100 μg/mL is required to decrease viability compared to the control.	[78]
	- Stem	- In vitro cytotoxic activity (MTT assay) on mouse lymphoma BS-24-1 and MoFir (EBV-transformed human B lymphocytes) cell lines.	- Ethanol extract inhibited proliferation of BS-24-1 and MoFir cells and reduced cell survival to ~30 and ~50%, respectively. - EO showed 87% and 40% against the same cell lines, respectively - β-Caryophyllene (**1.2.11**) isolated from *C. gileadensis* showe strong cytotoxic effect (85–90%) against both cell lines. - β-Caryophyllene induced apoptosis selectively in tumor cells via Caspase-3.	[34]
	- Stem bark	- In vitro cytotoxic activity on MCF-7 (breast) and HepG2 (liver) cell lines.	- Isolated prenylated flavonoids; comophoroside A (**3.7.6**) showed highest activity with IC_50_ of 8 and 12 µg/mL, respectively.	[46]
	- Stem bark	- In vitro cytotoxic activity on A549 (lung) and HELA (cervical) cell lines	- Methanol extract showed moderate anticancer properties; IC_50_ values of 24.5 and 24.9 µg/mL, respectively.	[85]
4. **Antidiabetic**	- Leaves and twigs	- Alloxan-induced diabetic rats. - Determination of fasting blood glucose, glycated haemoglobin HbAlc, and serum α-amylase.	- Both extracts significantly decreased serum fasting blood sugar and HbA1C levels. The aqueous extract the twigs was more efficient, but no significant increase in α-amylase levels.	[79]
	- Sap - Aerial parts	- Streptozotocin (STZ)-induced diabetes in mice, evaluating: - Random blood glucose levels - Glycated hemoglobin (HbA1c)	- Sap-treated group achieved normal blood glucose levels after 6 days of treatment, compared to 15 days for the aerial parts (methanol/acetone) extract. - Both groups showed significant decrease in HbA1c values vs. the untreated diabetic group. - Reduced inflammatory CD4^+^ and CD8^+^ cells while increasing T regulatory cells; reduced ET-1 and VEGF cardiovascular malfunction markers.	[86,87]
	- Stem bark	- Streptozotocin (STZ)-induced diabetes in rats - Serum blood glucose level (BGL), - α-Amylase and insulin level - Glibenclamide (GLC) as an antidiabetic standard	- *n*-Butanol fraction given to normal rats: No significant change in their (BGL) or α-amylase levels. - -BGLs reduced by 41.45% and α-amylase levels dropped by 25.00% compared to GLC (52.84% and 33.82%, respectively). - Insulin level significantly decreased in diabetic rats by 53.17% - Treatment with fraction +GLC: A significant increase in insulin level by - 44.91 and 63.55%, respectively, referring to diabetic rats.	[45]
5. **Wound healing**	- Leaves and branches	- Excision wound in mice (non-infected and *S. aureus*-infected). - Wound contraction percentage - Histopathological study.	- % Wound contraction by methanol extract was significantly higher than that of the control group. - Re-epithelization was higher than in the control group - Significant statistical differences in inflammatory cell infiltration, collagen fibres, re-epithelization and granulation tissue formation.	[49]
	- Fresh Stems	- Full-thickness skin excision in rats.	- 1% EO cream enhanced contraction (92.6% by day 20) and improved histological parameters. - Chloroform extract (cream 3%) significantly promoted faster contraction and shortened epithelialization time.	[36]
6. **Cardio-protective and vasorelaxant**	- Dehydroabietic acid (**1.3.1**) and sandaracopimaric acid (**1.3.5**), isolated from the resin.	- Pulmonary artery ring (male Wistar rats) - Measurement of NO Production in pulmonary artery endothelial cells (PAECs).	- Compounds **1.3.1** and **1.3.5** relaxed PE (phenylephrine) contracted pulmonary artery in a concentration-dependent manner. - Compound **1.3.1** increased nitric oxide (NO) production, along with the increased phosphorylation level of eNOS and Akt in endothelial cells.	[38]
	- Sap - Leaves	- STZ-induced diabetic mice and measurement of: - Cardiovascular integrity markers (adropin and NO). - Cardiovascular malfunction markers: AST, endothelin-1 (ET-1), and vascular endothelial growth factor (VEGF). - Cardiac enzyme markers: lactate dehydrogenase (LD), total creatine kinase (CK), creatine kinase-MB (CK-MB).	- Diabetic groups treated with extracts (methanol/acetone) showed: - Serum AST and ET-1 decreased significantly. - Adropin and NO increased (*p* < 0.01). - Significantly lowered LD, total CK, CK-MB, and VEGF levels than the untreated diabetic group (*p* < 0.01).	[86]
7. **Antihyperlipidemic**	- Leaves and twigs	- Alloxan-induced diabetic rat model, with measurement of: The triglycerides (TG), total cholesterol (TC), high density lipoprotein (HDL), low density lipoprotein (LDL) and the very low-density lipoprotein (VLDL).	- Both extracts decreased the TC, TG, LDL, and VLDL and significantly increased HDL levels compared to the positive control. - Twig aqueous extract was more efficient.	[79]
	- Sap - Leaves	- STZ-induced diabetic rat model, with measurement of: TG, TC, HDL, and LDL.	- The sap and acetone extract-treated groups had significantly lower levels of TG, TC, LDL and higher levels of HDL than the untreated diabetic group. - Sap-treated group achieved lower levels of TG and TC than the acetone extract group.	[86]
	- Stem bark	- STZ-induced diabetic rats	- *n*-Butanol fraction decreased TC (17.18%), TG (14.73%), and LDL (43.13%); significantly increased HDL (39.57%).	[45]
8. **Gastroprotective and anti-ulcer activity**	- Aerial parts	- Acute gastric ulcer models in rats induced by: - 80% ethanol, 0.2 M NaOH, and 25% NaCl - Hypothermic restraint stress - Pyloric ligation (Shay rat model) - Indomethacin. - Histopathological study.	- Ethanol extract (250 and 500 mg/kg) administered orally (intraperitoneally in the Shay rat model) showed dose-dependent ulcer protective effects in all ulcer models. - Extract offered protection against ethanol-induced depletion of stomach wall mucus and reduction in nonprotein sulfhydryl (NP-SH) concentration.	[74]
9. **Hepatoprotective**	- Stem bark	- STZ-induced diabetic rats, measuring the level of liver function enzymes: AST, ALT, and ALP.	- *n*-Butanol improved endogenous defenses; GSH increased by 82.51% and SOD by 49.03%. - Decrease in: AST by 30.21%, ALT by 48.73%, ALP by 26.10%	[45]
	- Aerial parts	- CCI_4_-induced acute liver injury in Wistar albino rats, measuring: AST, ALT, ALP, and serum bilirubin concentrations. - Histological study. - Measurement of phenobarbital-induced sleeping time.	- Ethanol extract (250 and 500 mg/kg) exhibited a significant reduction in levels of AST, ALT, ALP, and serum bilirubin concentrations. - Shortened phenobarbital-induced sleeping time at a dose of 500 mg/kg, indicating restoration of the hepatic drug-metabolizing system. - Histological observations supported the results obtained from liver enzyme assays.	[72]
	- Leaves - Twigs	- Alloxan-induced diabetic hypercholesterolemic rats, with measurement of: - Alanine aminotransferase (ALT). - Aspartate aminotransferase (AST). - Alkaline phosphatase (ALP). - Gamma-glutamyl transferase (GGT). - Histopathological study.	- Aqueous extracts restored the levels of liver function enzymes nearly to normal. - Twig extract was more efficient than leaf extract in restoring liver function enzymes and hepatic tissue structure to normal. - Histopathological study shows: - Leaf aqueous extract: Near-normal hepatic tissues - Twig aqueous extract: a normal hepatic tissue	[79]
	- Bark	- Diethylnitrosamine (DEN)-induced injury promoted by phenobarbitone in rats.	- Methanol extract significantly reduced liver enzymes: AST, ALT, and ALP. - Histopathological studies showed marked improvement in steatosis and necrosis.	[73]
10. **Fertility-enhancing**	- Sap - Aerial parts	- STZ-induced diabetic rats, with measurement of: - Body and testicular weights. - Testicular homogenate antioxidant and oxidative stress parameters. - Semen analysis (sperm count, morphology, motility) - Measurement of serum testosterone, follicle-stimulating hormone (FSH), luteinizing hormone (LH), prolactin, endothelin, adropin, and NO levels. - Testicular immunohistochemistry analysis.	- Compared to the untreated groups: - The body weight of groups treated with extracts (methanol/acetone) was significantly higher. - Sap groups significantly increased testicular weight and higher sperm count than the untreated group (*p* < 0.05). - Groups treated with extracts had greater testosterone, NO, adropin, and NOS immunoreactivity. - Significantly induced GSH, GSH-Px, and SOD activity in the testes. - Restored normal seminiferous tubule architecture and increased NOS immunoreactivity.	[86]
11. **Antigenotoxic**	- Shoots	- CCl_4_-induced genotoxicity and reproductive damage in mice in male SWR mice, with assessment of: - Chromosomal abnormalities in bone marrow - DNA fragmentation assay in hepatocytes was calorimetrically detected by CCl_4_ calorimetrically detected. - Spermatocytes and sperm shape abnormalities	- Dichloromethane extract significantly reduced the proportion of chromosomal abnormalities in bone marrow cells, DNA fragmentation in hepatocytes, and sperm shape anomalies induced by CCl_4_ after 7 days of treatment with the low dose (200 mg/kg b.w.), and high dose (200 mg/kg b.w.) of the tested extracts, respectively. - DNA fragmentation in hepatocytes by 40.68, 52.41% - Chromosome irregularities in spermatocytes by 45.9 and 54.05%	[32]
	- Stem bark	- STZ-induced diabetes in Wistar rats - DNA damage was evaluated by the ratio of tail DNA content/the whole cellular DNA content. using the Comet assay. Noticing the following criteria: - DNA tail lengths - % of tailed DNA	- *n*-Butanol fraction reduced % of tailed DNA from 13% in the untreated diabetic group to 11%, compared to 9% for the Glibenclamide group. - Restored the proportion of healthy untailed DNA from 87% to 89%, compared to 91% for Glibenclamide treated group. - Reducing DNA tail lengths and tail moments.	[45]
12. **Immunomodulatory**	- Sap - Leaves and branches	- STZ-induced diabetic male mice model, assessing CD3^+^, CD4^+^, CD8^+^, and CD25^+^ T-lymphocytes (Flow cytometry).	- Sap and acetone extract reduced the total lymphocytes - Reduced inflammatory CD3^+^, CD4^+^, and CD8^+^ cells. - Significantly increased regulatory CD4^+^ CD25^+^ and CD8^+^ CD25^+^ T-cells. - Sap showed more efficacy compared to its acetone extract	[86,87]
	- Leaves - Twigs	- Alloxan-induced diabetic hypercholesterolemic rats. - Serum immunoglobulins (IgA, IgM, IgE, and IgG)	- Aqueous extracts significantly restored the immunoglobulins IgA, IgM, and IgG (previously high), and IgE (previously low) to nearly normal levels. - The twig aqueous extract was more efficient.	[79]
13. **Anti-aging**	- Leaves	- Replicative lifespan assay using the K6001 yeast strain	- USE and HDE increased yeast average lifespan at a concentration of 30 μg/mL (increased to 9.15 ± 0.62 generations vs. 7.55 ± 0.48 in control).	[78]
14. **Analgesic**	- Aerial parts	- Acetic acid-induced writhing in mice - Tail flick test	- Ethanol extract significantly reduces writhes induced by acetic acid. - Significantly increased the tail flick latency to the nociceptive stimuli.	[76]
	- Aerial parts	- Acetic acid-induced writhing in mice - Hot plate in mice - Formalin paw lick techniques. - Test the analgesic effect of a combination of reduced doses of the extract and diclofenac.	- Methanol extract showed a stronger inhibition of writhing compared to diclofenac, and the dose of 500 mg/kg completely inhibited the writhing response. - In the hot plate test, the extract combined with diclofenac exhibited significant prolongation of reaction time compared to diclofenac alone. - The extract (500 mg/kg) significantly shortens the time of licking compared to diclofenac at both phases. - The combination resulted in synergistic potentiation of the analgesic effect.	[82]
15. **Antipyretic**	- Aerial parts	- A subcutaneous injection of 20 mL kg^−1^ of a 20% aqueous suspension of brewer’s yeast for mice.	- Ethanol extract caused a dose- and time-dependent decrease in yeast-induced hyperthermia measured rectally.	[76]
16. **Diuretic, kidney protective, and antihyperuricemic**	- Aerial parts	- Albino Wistar rats and Swiss mice - Measurement of urine volumes. - Urine analysis for Na^+^, K^+^, and Ca^2+^ content.	- Ethanol extracts 250 and 500 mg kg^−1^ b.w.) caused marked diuresis. - No significant change in Na^+^, K^+^, and Ca^2+^ ions excretion.	[76]
	- Leaves - Twigs	- Urea, creatinine, and uric acid levels - Histopathological study	- Aqueous extracts significantly ameliorated all the serum kidney index levels, with the twigs extract being more effective. - Nearly restored normal renal tissues of groups treated with both aqueous extracts.	[79]
	- Aerial parts	- Xanthin oxidase inhibitory test	- Ethanol extract showed weak xanthine oxidase activity (IC_50_ 251.2 µg/mL) compared to allopurinol (0.41 µg/mL).	[11]
17. **Anticoagulant**	- Sap - Leaves and branches	- Mouse coagulation profiles, prothrombin time (PT), activated partial thromboplastin time (aPTT), international normalized ratio (INR), naïve mice.	- Sap, methanol, and acetone extracts prolonged PT, aPTT, and bleeding time in naïve mice more than heparin and aspirin.	[88]
18. **Antibacterial and antibiofilm**	- Leaves and seeds, callus, and cell suspension culture	- Antimicrobial assays with two methods: Disc diffusion assay and minimum inhibitory concentration (MIC) against: - *S. aureus* ATCC-25923 - Bacillus subtilis ATCC-6633 - *S. epidermidis* ATCC-12228 - *Salmonella* spp. - *Escherichia coli* - *Erwinia carotovora*	- Methanol extract caused a selective antimicrobial effect. - No effect against *E. coli*, *E. carotovora*, and *K. pneumonia*. - *S. aureus* was the most sensitive with all extracts except for the callus. - The leaf extract showed the highest activity (IZD = 2.5 cm). - The cell suspension was effective for *S. epidermidis* and *S. aureus*. - The callus extract was not effective on all tested microbes.	[23]
	- Aerial parts	- Agar well diffusion assay for: - Gram-positive: *S. aureus* ATCC 6538. - Gram-negative: *E. coli* ATCC 8739.	- Ethanol extract showed moderate activity against *S. aureus* (15 mm zone), but no activity against *E. coli*. - EO exhibited strong inhibition against *S. aureus* (20 mm zone) and *E. coli* (10 mm zone).	[11]
	- Leaves and branches	- In vivo mouse excision wound model and inoculum of *S. aureus* suspension containing 10^6^ CFU/mL. - −3 mg/g of gentamicin used as reference.	- Methanol extract decreased colonization of the infected wounds (CFU) counts in *S. aureus*-infected wounds and accelerated wound contraction compared to the control group.	[49]
	- Leaves and branches	- In vitro and in vivo against MRSA in mice model.	- Methanol extract showed an IZD of 7 mm for MRSA and 3 mm for *P. aeruginosa* and reduced mice mortality by nearly 50%.	[89]
	- Leaves and stems	- Crystal violet method for (detection of biofilm formation) - Phenol-sulphonic acid method to measure exopolysaccharide (EPS) contents. - Mouse wound model, inoculation with *S. aureus* (10^6^ CFU/mL). - Colony-Forming Unit (CFU) Count - Bacteria: Multidrug-resistant *E. coli*, *A. baumannii*, *Listeria monocytogenes*, *Serratia marcescens*, *K. pneumonia*, *S. aureus*, *P. aeruginosa*.	- No significant differences between leaves and stems extracts. - Strongest effect for methanol, followed by ethyl acetate and chloroform, then hot water extracts. - The highest biofilm inhibition was against *K. pneumonia* (highest EPS content of 0.29 ± 0.04 μg/mg of cells), which was reduced by 39% after using the plant extract. - There is a synergistic effect between the *C. gileadensis* extract and some antibiotics, especially Amoxicillin, Polymixin B, and Tetracycline.	[81]
	- Branches	- In vitro study for extracts on 1- and 3-week-old oral anaerobic multispecies biofilms and to compare them to 2% chlorhexidine (CHX). - Biofilm discs are exposed to 1 or 3 min for the following: - Extract of different concentrations in DMSO and water: 1 mg/mL water, 0.1 mg/mL water, 1 mg/mL 0.5% DMSO, 0.1 mg/mL 0.5% DMSO - Water, 2% CHX and 0.5% DMSO. - Discs were stained with a viability stain and scanned under a confocal laser scanning microscope. - The % of dead bacteria.	- Exposure of the biofilms to methanol extract (1 mg/mL in water) had the highest percentage of dead bacteria (42.13–46.67% ± 4.5–8.0) among all the groups, regardless of the age of the biofilm and the time of exposure to the agents (1 or 3 min), and the difference was statistically significant (*p* < 0.05). - Extract 1 mg/mL in water killed significantly more bacteria in oral anaerobic multispecies biofilm than 2% CHX.	[90]
	- Fresh stems; isolated *ent*-verticillane-type diterpenes	- Broth dilution method (MIC)	- (9*S*,10*S*)-*Ent*-9,10-epoxyverticillol was most active against *K. pneumoniae* (MIC 0.025 mg/mL).	[39]
	- Aerial parts	- In vitro antimicrobial panel	- EO exhibited activity against *B. subtilis*, *S. aureus*, and *Mycobacterium intracellulare*.	[33]
	- Stem peel and leaves	- Agar well diffusion and MIC	- Stem peel methanolic extract more active than leaves; strongest inhibition against *E. coli* (18 mm zone); MICs ranged from 128 to 256 µg/mL.	[80]
	- Bark	- Agar cup plate method	- Gel formulation of methanol extract showed higher antibacterial activity (15.7 mm IZD) against *S. aureus* than the cream formulation and standard cetrimide cream.	[70]
	- Branches	- Confocal laser scanning microscopy (CLSM)	- Aqueous extract (1 mg/mL) significantly killed more bacteria in oral anaerobic multispecies biofilms than 2% chlorhexidine.	[90]
	- Bark	- Modified agar diffusion method	- Methanol extract demonstrated antibacterial activity against *S. aureus*, *P. aeruginosa*, and *K. pneumoniae* and was safe in mice up to 5 g/kg.	[85]
19. **Antiviral**	- Leaves	- The plaque reduction assay (Vero cells), for enveloped viruses: HSV-2 and RSV-B. - The MTT assay (HEp-2 cells), for nonenveloped viruses: CVB-3 and ADV-5. - Bio-guided assays for isolation of the active compound involving TLC.	- The methanol extract showed antiviral activity against the enveloped viruses (HSV-2 and RSV-B) with IC_50_ = 20 µg/mL and selectivity index (SI) > 10. - This extract was inactive against the non-enveloped viruses (CVB-3 and ADV-5). - The active compound was identified as guggulsterone.	[44]
	- Bark	- The antiviral activity was - determined by the inhibition of the cytopathic effect compared to the control. - Tested viruses: HAV-0, Coxsacki, HSV-1, and HSV-2.	- Methanol extract was totally ineffective against all tested viruses.	[91]
20. **Antifungal**	- Callus and ex vitro shoots	- Agar well diffusion method used with 25, 50, 75 and 100 μL against six fungal species: - *Aspergillus nidulans*, *A. niger mutant black*, *Penicillium italicum*, *P. chrysogenum*, *Phytophthora infestans* (Location 1 and 2, L1 and L2)	- Both extracts (methanol) showed considerable inhibition effects on the tested fungi. - The highest activity was for callus extract against *P. infestans* L1 (IZD = 42.3 mm), using 100 µL. - Callus was more effective than ex vitro shoots against *A. nidulans* (IZD = 24.6 mm at 100 µL). - Ex vitro shoots extract inhibited all fungal species except *A. nidulans*. - Ex vitro shoots extract was highly effective against *P. chrysogenum* (IZD = 33.3 mm) compared to callus (11.1 mm).	[92]
	- Leaves	- Disc diffusion assay and MIC - *C. albicans* ATCC-10231	- Methanol extract effectively inhibited the growth of *C. albicans* (IZD = 18 mm).	[23]
	- Fresh and dry stem extracts collected from different locations	- Well-agar diffusion method - *C. albicans*	- Significant variation in the anticandidal inhibition properties of the tested extracts due to solvent type (methanol, acetone, hot water, chloroform, dichloromethane, diethyl ether and cyclohexane), plant locations, and form of plant extract, either fresh or dry.	[37]
	- Bark	- A modified agar diffusion method, yeast (*Candida* species) using (itraconazole and voriconazole) as references.	- Methanol extract showed activity against *Candida* species. - It was effective against strains resistant to itraconazole and voriconazole.	[85]
	- Fresh aerial parts	- Broth microdilution susceptibility assay (IC_50_) - Fungal panel: - *Candida glabrata* - *Candida krusei* - *Cryptococcus neoformans*	- EO exhibited weak activity against *C. glabrata* (IC_50_ = 80 µg/mL), *C. krusei* (90 µg/mL), and *C. neoformans* (150 µg/mL).	[33]
	- Fresh stems	- Agar well diffusion and broth dilution (MIC) methods	- Chloroform extract was identified as the most active fraction against *C. albicans* during fractionation (MIC = 125 µg/mL).	[39]

**Table 5:** Toxicological studies reported for *C. gileadensis*.

Extract Type	Animals/Test Model	Route	Doses	Result	Ref.
Bark extract (methanol)	- White albino mice with observation for 24 h. - LD_50_ test.	- Oral	- Extracts were dissolved in water at doses of 100, 1000, 2500, 4000, 5000 mg/kg b.wt.	- The extract was safe up to 5000 mg/kg with no acute toxic signs or mortality, except reversible reduction in motor activity at doses of 2500, 4000, and 5000 mg/kg.	[85]
Bark extract (butanol fraction)	- Male Wistar albino rats/observed for 15 days.	- Oral	- 50, 100, 200 mg/kg b.wt.	- No mortality	[45]
Leaf extract (methanol)	- Mice observed for 24 h.	- Subcutaneous injection	- 100, 1000, 2000, 3000, 4000 mg/kg b.wt.	- No mortality or toxicity signs.	[89]
Aerial parts extract (methanol)	- Rats observed during the 14 days. - Histological examination of vital organs (brain, heart, kidney, stomach and spleen). - Hematological and coagulation parameters. - Biochemical parameters: Urea, Creatinine, Glucose, Lipid profile: Cholesterol, LDL, HDL and TG, Liver function tests (AST and ALT).	- Oral (gavage)	- 250, 500 and 1000 mg/kg b.wt./day	- No mortality and no signs of toxicity survived active and healthy. No significant difference in the body weight of the rats. - No histological changes, insignificant changes compared to the control group.	[127]
			- >2000 mg/kg b.wt./day	- No mortality or toxicity signs. - A remarkable observation: Sedation with weak response to stimuli (sounds or thrust). - Parameters: no significant harmful outcome	

## Data Availability

No new data were created or analyzed in this study. Data sharing is not applicable to this article.

## Abbreviations

The following abbreviations are used in this manuscript:

ADME	Absorption, distribution, metabolism, and excretion	INR	International Normalized Ratio
ALP	Alkaline phosphatase	IUCN	International Union for Conservation of Nature
ALT	Alanine aminotransferase	IZD	Inhibition zone diameter
aPTT	Activated partial thromboplastin time	LC-MS	Liquid chromatography-mass spectrometry
AST	Aspartate aminotransferase	LC-MS/MS	Liquid chromatography with tandem mass spectrometry
BCB	β-carotene bleaching	LD	Lactate dehydrogenase
BGL	Blood glucose level	LD_50_	Median lethal dose
CAT	Catalase	LDL	Low-density lipoprotein
CD	Cluster of differentiation	MIC	Minimum inhibitory concentration
CFU	Colony-forming unit	MRSA	Methicillin-resistant *Staphylococcus aureus*
CHX	Chlorhexidine	NMR	Nuclear magnetic resonance
CK-MB	Creatine kinase-MB	NO	Nitric oxide
COX	Cyclooxygenase	NOS	Nitric oxide synthase
COX-1	Cyclooxygenase-1	NP-SH	Nonprotein sulfhydryls
DEN	Diethylnitrosamine	NSAIDs	Non-steroidal anti-inflammatory drugs
DM	Dry matter	PDA	Photodiode array
DMSO	Dimethyl sulfoxide	PI3K/Akt-eNOS	Phosphoinositide 3-kinase/Protein Kinase B—endothelial Nitric Oxide Synthase
DPPH	2,2-diphenyl-1-picrylhydrazyl	PT	Prothrombin time
EBV	Epstein–Barr virus	PXR	Pregnane X receptor
EO	Essential oil	ROS	Reactive oxygen species
EPS	Exopolysaccharide	SOD	Superoxide dismutase
ESI-MS	Electrospray ionization mass spectrometry	STZ	Streptozotocin
ET-1	Endothelin-1	TC	Total cholesterol
FRAP	Ferric reducing antioxidant power	TG	Triglycerides
FXR	Farnesoid X receptor	TNF-α	Tumour Necrosis Factor-alpha
GC-MS	Gas chromatography-mass spectrometry	TPC	Total phenolic content
GPx/GSH-Px	Glutathione peroxidase	Treg	T-regulatory cells
GSH	Reduced glutathione	UPLC-qTOF-MS	Ultra Performance Liquid Chromatography—Quadrupole Time-of-Flight Mass Spectrometry
GST	Glutathione S-transferase	USE	Ultrasonic-assisted extraction
HbA1c	Glycated hemoglobin	UV	Ultraviolet
HDL	High-density lipoprotein	VEGF	Vascular endothelial growth factor
HPLC	High-performance liquid chromatography	VLDL	Very low-density lipoprotein
HS-SPME	Headspace solid phase microextraction	XO	Xanthine oxidase
